# Design of Novel Pyrene-Bodipy Dyads: Synthesis, Characterization, Optical Properties, and FRET Studies

**DOI:** 10.3390/molecules23092289

**Published:** 2018-09-07

**Authors:** Pasquale Porcu, Mireille Vonlanthen, Israel González-Méndez, Andrea Ruiu, Ernesto Rivera

**Affiliations:** Instituto de Investigaciones en Materiales, Universidad Nacional Autónoma de México, Circuito Exterior, Ciudad Universitaria, Mexico City CP 04510, Mexico; paskpo89@gmail.com (P.P.); mireille.vonlanthen@gmail.com (M.V.); israelgonzalezme@gmail.com (I.G.-M.); andrea1.ruiu@gmail.com (A.R.)

**Keywords:** bodipy, pyrene, fluorescence resonance energy transfer (FRET)

## Abstract

A new series of dendronized bodipys containing pyrene units was synthesized and characterized. Their optical and photophysical properties were determined by absorption and fluorescence spectroscopy. This series includes three different compounds. The first one has an anisole group linked to the bodipy unit, which was used as the reference compound. In the second, the bodipy core is linked to a zero generation dendron with one pyrene unit. The third compound contains a first generation Fréchet-type dendron bearing two pyrene units. In this work, the combination pyrene-bodipy was selected as the donor-acceptor pair for this fluorescence resonance energy transfer (FRET) study. Doubtless, these two chromophores exhibit high quantum yields, high extinction coefficients, and both their excitation and emission wavelengths are located in the visible region. This report presents a FRET study of a novel series of pyrene-bodipy dendritic molecules bearing flexible spacers. We demonstrated via spectroscopic studies that FRET phenomena occur in these dyads.

## 1. Introduction

The development of new photoactive compounds bearing donor-acceptor groups has very relevant in materials science [1,2,3]. In order to understand the energy transfer process that occurs in natural chlorins, many chemists have developed and designed more efficient materials for light energy conversion [3,4,5]. The fluorescence resonance energy transfer (FRET) phenomenon is a non-radiative process that includes the energy transfer between two molecules or two different groups in a molecule [6,7,8,9,10,11,12]. One of the molecules or groups is in the ground state (acceptor), whereas the other is in the first excited state (donor). The FRET phenomenon occurs via a Förster mechanism when the emission spectra of the donor group overlap, partially or totally, with the absorption spectra of the acceptor.

Bodipy (4,4-difluoro-4-bora-3a,4a-diaza-s-indacene) dyes have attracted the attention of many research groups due to its special photophysical properties. They present a strong ultraviolet (UV) absorption, relatively sharp fluorescence peaks with high quantum yields, and very good photostability [13,14,15,16,17]. Furthermore, they are relatively insensitive to the polarity and pH of the environment, and are reasonably stable to physiological conditions, showing high solubility in many organic solvents [15,18]. The main aspect that make bodipy dyes photophysically suitable is the tunable emission/absorption by functionalization [19,20].

Notably, pyrene possesses outstanding photophysical properties, such as high quantum yield, long lifetime, and ease of formation of excimers [21,22,23,24,25,26,27]. For these reasons, our group focused our attention on the study of dendritic molecules bearing pyrene as the donor group and different acceptor groups, such as porphyrin, fullerene, and bispyridinium organometallic complexes, among others [24,28,29].

We selected pyrene as the donor and bodipy as the acceptor chromophores in order to carry out a FRET study, since the emission spectrum of pyrene significantly overlaps the absorption spectrum of bodipy. A few reports are presented in the literature using this pair of chromophores, where they are directly linked or are attached via a short rigid spacer [19,30,31,32,33]. The idea of this work was to connect both moieties through alkylic flexible chains and Fréchet type dendrons, allowing these chromophores to encounter one another, and for the orientation of their dipole moments to occur at a distance not longer than 10 nm, which are the conditions for the FRET phenomenon to occur [34]. Moreover the use of a Fréchet type dendron allowed us to obtain a molecular antenna, where more than one donor group can be attached to only one acceptor.

## 2. Results and Discussion

### 2.1. Synthesis of the Pyrene-Bodipy Compounds

**Scheme 1:**
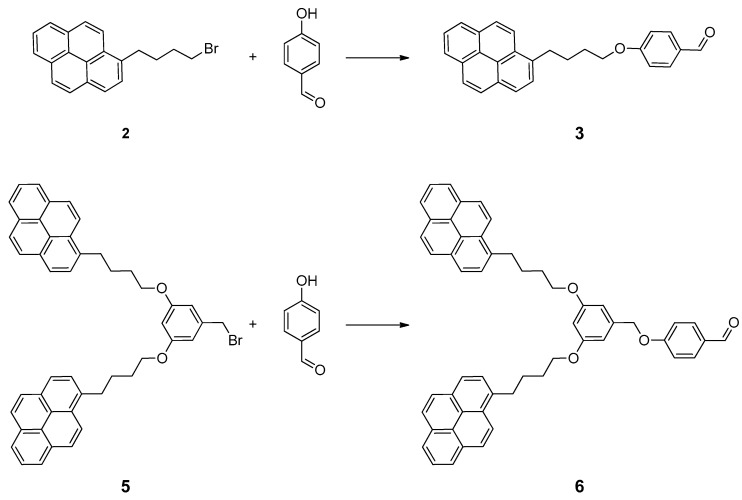
Synthetic route for the aldehydic compounds **3** and **6**. Reaction conditions: K_2_CO_3_, 18-crown-6, dimethylformamide (DMF), 110 °C, and four hours (yield 86% for **3** and 79% for **6**).

The synthesis of pyrene-labeled dendrons **3** and **6** was carried out following the classical convergent method shown in Scheme 1. The synthetic route of the bromide precursors **2** and **5** was previously reported by our research group [24]. A nucleophilic substitution of compounds **2** or **5** with p-hydroxybenzaldehyde was carried out using K_2_CO_3_ as a base and 18-crown-6 as a catalyst. After purification by column chromatography, the desired compounds **3** and **6** were obtained as white viscous solids with yields of about 80%. Proton nuclear magnetic resonance (^1^H NMR), carbon NMR (^13^C NMR), and electrospray ionization (ESI) mass spectroscopy confirmed the chemical structures of compounds **3** and **6**.

**Scheme 2:**
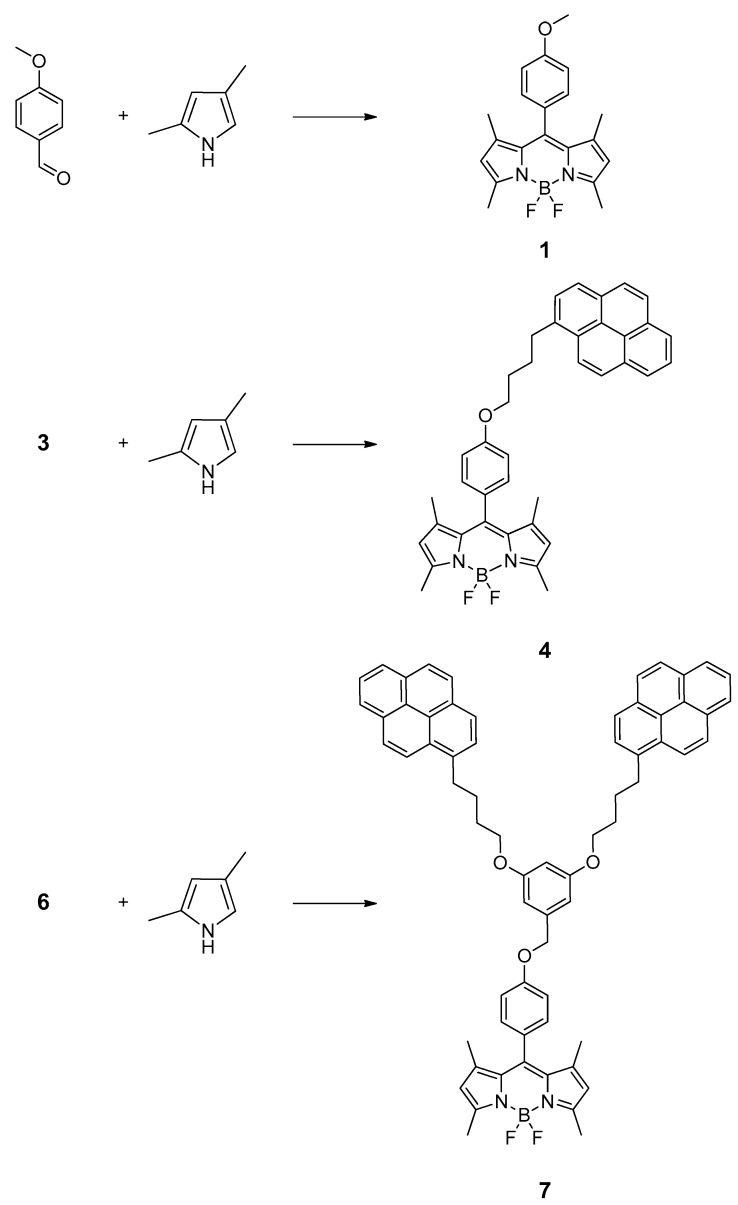
Synthesis of the pyrene-labeled bodipy compounds **1**, **4**, and **7**. Reaction conditions (1) trifluoroacetic acid (TFA), CH_2_Cl_2_, 4 h, t.a.; (2) DDQ, 45 min; (3) N(Et)_3_, BF_3_O(Et)_2_, 15 min. Yield 46% for **1**, 38% for **4**, and 47% for **7**.

The aim of the second part of the synthesis was to obtain the final compounds bearing bodipy and pyrene units. In order to have a reference, compound **1** was synthesized according to a procedure previously reported in the literature [14]. Generally, the synthesis of bodipy compounds starts with a condensation between an aldehydic compound and two equivalents of pyrrole in the presence of trifluoroacetic acid (TFA) as catalyst [13,16,35]. In our case, compounds **3** and **6** were employed to generate the new series (Scheme 2). After four hours, an oxidation in presence of 2,3-dichloro-5,6-dicyano-1,4-benzoquinone (DDQ) was performed, in order to oxidize position 8. Finally, the bodipy compounds were obtained by addition of NEt_3_ as catalyst and BF_3_O(Et)_2_. After purification by column chromatography, compounds **1**, **4**, and **7** were obtained as red solids with a yield of about 50%. ^1^H NMR, ^13^C NMR, ESI, and Matrix-Assisted Laser Desorption/Ionization Time-of-Flight (MALDI-TOF) mass spectroscopy confirmed the chemical structures of the compounds.

### 2.2. Optical and Photophysical Properties of the Pyrene-Labeled Bodipy Compounds

**Figure 1:**
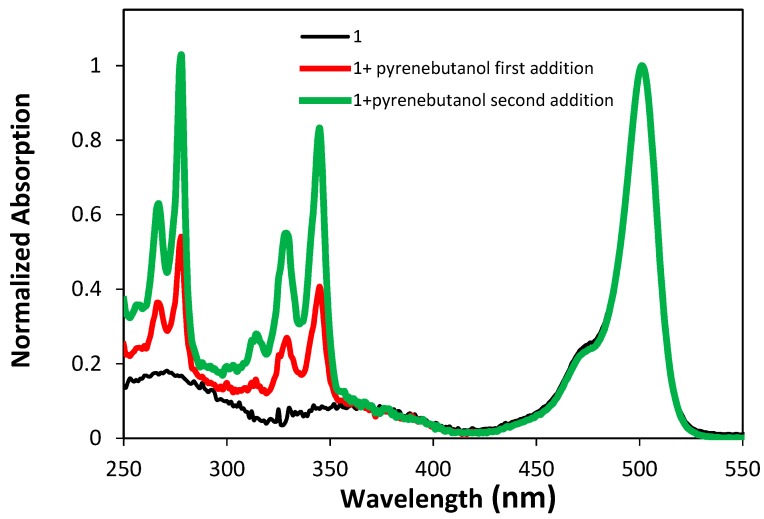
Absorption spectra for the compound **1** (1.2 × 10^−5^ M), a mixture of **1** (1.2 × 10^−5^ M) and 1-pyrenebutanol at 1.2 × 10^−5^ M and 2.4 × 10^−5^ M.

The optical and photophysical properties of the bodipy analogue **1**, pyrene-labeled aldehyde precursors **3** and **6,** as well as those of the pyrene-labeled bodipy compounds **4** and **7**, were evaluated in THF solution. The first step in our work was to analyze if the pyrene and bodipy chromophores were interacting in the ground state when they are not linked covalently to each other. Therefore, a solution compound **1** (1.2 × 10^−5^ M) was prepared. Firstly, the absorption spectrum of compound **1** was recorded; it corresponded to an absorption value of 1 at 501 nm. Then, a solution of 1-pyrenebutanol (2.4 × 10^−5^ M) was prepared using the solution of compound **1** as a solvent in order to maintain the concentration of **1** constant. One consecutive dilution of 1-pyrenebutanol (1.2 × 10^−5^ M) was performed. As a result, the absorption spectra demonstrated a regular increase in the absorption band corresponding to the pyrene moiety at 344 nm, whereas the part of the spectra corresponding to the absorption of the bodipy reference compound at 501 nm remained unchanged (Figure 1). Therefore, we concluded that there are no interactions between these two chromophores when they are not linked in a chemical structure.

Compound **1** presented the typical absorption band of a bodipy chromophore [31,36,37]. The molar absorption coefficient for the most intense band at 501 nm was calculated to be 87,000 M^−1^ cm^−1^. Furthermore, an interesting band between 320 and 400 nm was also observed. This absorption band was due to *n*-π* of the free electron pair in the methoxy group [38]. We calculated the molar absorption coefficient, with a value of about 6400 M^−1^ cm^−1^ at 362 nm in THF solution. 

**Figure 2:**
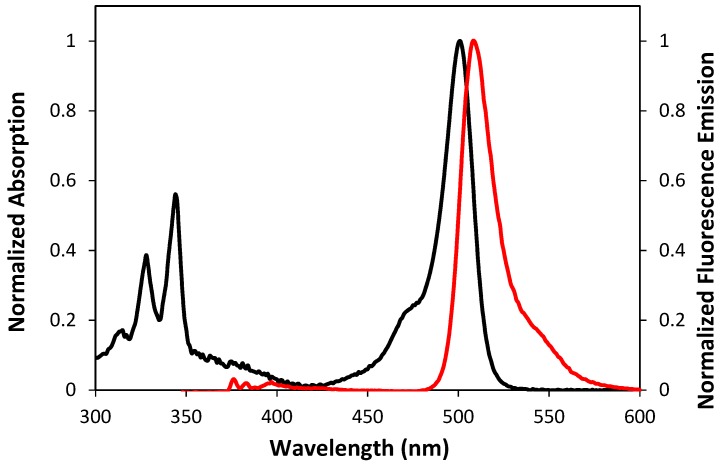
Absorption (black) and emission (red) spectra for the compound **4**.

**Figure 3:**
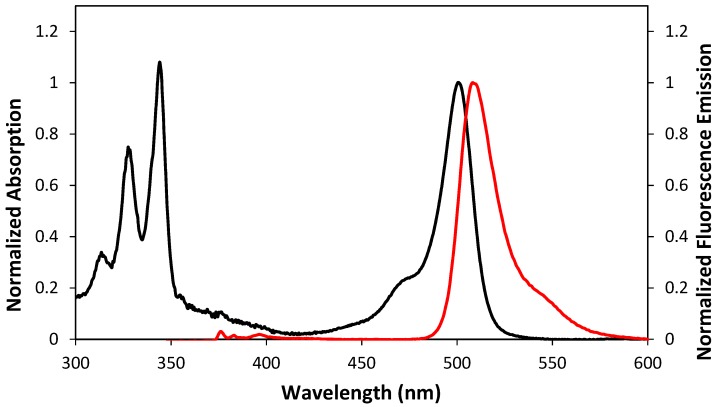
Absorption (black) and emission (red) spectra for compound **7**.

The absorption spectra of compound **4** presented the typical pyrene transition band at 344 nm due to the S_0_ → S_2_ transition [23,39] and the bodipy band at 501 nm for the S_0_ → S_1_ transition [31,35,36,40] (Figure 2). The spectra showed that the previously characterized band (320–400 nm) was preserved. We calculated the molar absorption coefficient for the two most intense bands at 344 and 501 nm (Table 1) in THF solution. Compound **7** showed higher pyrene absorption in comparison with compound **4** due to the presence of two pyrene units (Figure 3). The absorption coefficients were calculated at the same wavelengths (Table 1).

The fluorescence spectra of compounds **3**, **4**, **6**, and **7** were measured in THF solution at room temperature with a concentration corresponding to an absorption of about 0.03 at excitation wavelength (344 nm). Compound **3** showed the typical monomer emission band of the pyrene moiety, whereas the emission spectra of compound **6** showed monomer as well as excimer emission, due to the presence of two pyrene units in the same molecule (Supporting Information Figures S2 and S3).

Compound **4** showed that the pyrene emission between 360 and 480 nm was almost totally quenched and presented a strong emission band corresponding to the bodipy unit at 509 nm (Figure 2). This is due to the presence of a FRET phenomenon. The same behavior was observed in compound **7**, whose emission spectrum showed the same characteristics even though there are two pyrenes units in the same molecule (Figure 3). 

The possibility that a FRET process occurs between the pyrene donor and the bodipy acceptor was evaluated through the determination of the Förster radius (R_0_) for these pair of chromophores using the following equation:

R_0_ = 9790 × (*k*^2^ × *n*^−4^*× φ_d_ × J*)^1/6^ (1)

where *k* is the orientation factor (2/3), *n* is the refractive index of the solvent (THF 1.4050), *φ* is the fluorescence quantum yield of the 1-pyrenebuthanol (value), and *J* is the overlap integral of the two chromophores, calculated using the following equation [40]:

$$J(\lambda )=\int_{0}^{\infty }f_{D}(\lambda )\varepsilon_{A}(\lambda )\lambda^{4}d\lambda$$

where *f_D_* is the donor emission spectrum that was normalized to its area, *ε_A_* is the molar absorption coefficient of the acceptor, and *λ* is the wavelength. 

As a result, the R_0_ value for this pair of chromophores in THF solution was calculated to be 2.208 nm. Considering the flexibility of the chemical structures, it is reasonable to estimate that, in solution, the two parts of the molecule can be brought closer together than the calculated Förster radius, and therefore the FRET phenomenon can easily occur.

The quantum yields of compounds **3**, **4**, **6**, and **7** were calculated (Table 1). All the experiments were carried out in triplicate. In the case of compounds **3** and **6**, due to their sensibility to oxygen, degassed solutions were required. The emission of the bodipy compounds **4** and **7** was not significantly affected by oxygen.

The difference in the quantum yields of pyrene in the dendrons and pyrene in the final compounds was due to the FRET phenomenon, which totally quenched the emission of this chromophore. When the excitation was performed at 344 nm, the values of the quantum yields showed that the FRET phenomenon occurs for compounds **4** and **7**. The bodipy part received all the energy from the pyrene transition S_2_ → S_0_ and subsequently emitted this energy at 510 nm. Particularly, in the case of compound **1**, a lower quantum yield was found at this wavelength confirming that the FRET phenomenon occurred. The quantum yields, calculated at 475 nm, exhibited comparable values to the one reported in the literature for a similar bodipy [17]. Compounds **4** and **7** showed a high FRET efficiency. Definitively, this study showed that between bodipy and pyrene FRET optimally occurs.

## 3. Materials and Methods 

### 3.1. General Notes

Boron trifluoride diethyl etherate (BF_3_O(C_2_H_5_)_2_) and 2,4-dimetylpyrrole were freshly distilled under reduced pressure before use. The solvents employed in the reaction were purified by distillation in the presence of drying reagents (metallic sodium for THF or CaH_2_ for dichloromethane). All the synthetic procedures were carried out under an argon atmosphere. The purification of the products was performed by column chromatography in silica gel and the elution was checked by thin layer chromatography (TLC). All intermediates and final compounds were characterized by ^1^H NMR and ^13^C NMR spectroscopies and MALDI-TOF mass spectrometry. NMR spectra were carried out on a Bruker Avance 400 MHz, operating at 400 MHz and 100 MHz for ^1^H and ^13^C, respectively. Samples were dissolved in deuterated chloroform. MALDI-TOF mass spectra were recorded using dithranol as matrix on a Bruker Daltonics Flex Analysis. UV-Visible (UV-Vis) spectra were carried out on a Unicam UV300 spectrophotometer using quartz cells with a width of 1 cm and THF (HPLC grade) as the solvent. Fluorescence spectra were recorded on a Fluorolog 3 spectrophotometer from Horiba with a xenon lamp as light source. The slit width for excitation and emission were defined to 1 nm. 

### 3.2. Synthetic Procedures

The synthesis of all the intermediates and final products is described in this section. For the assignment of the signals, please see the Supporting Information. 

*5,5-difluoro-10-(4-methoxyphenyl)-1,3,7,9-tetramethyl-5H-dipyrrolo[1,2-c:2′,1′-f][1,3,2]diazaborinin-4-ium-5-uide* (**1**). Compound **1** was synthesized according the procedure previously reported in the literature [14]. The product was obtained as a red solid (394.5 mg, 1.1 mmol). Yield: 46%. ^1^H NMR (CDCl_3_, 400 MHz): 7.15 (m, 2H, H^15,17^), 6.99 (m, 2H, H^14,18^), 5.97 (s, 2H, H^8,11^), 3.87 (s, 3H, H^20^), 2.55 (s, 6H, H^24,26^), 1.43 (s, 6H, H^23,25^). ^13^C NMR (CDCl_3_, 100 MHz): 160.1 (C^16^), 155.3 (C^6^), 143.2 (C^13^), 141.9 (C^9^,C^19^), 131.9 (C^5^,C^1^), 129.2 (C^14^,C^18^), 127.1 (C^7^,C^12^), 121.1 (C^11^,C^8^), 114.5 (C^15^,C^17^), 55.3 (C^20^), 14.6 (C^23^,C^24^,C^25^,C^26^). ESI: *m/z* calculated for C_20_H_21_BF_2_NO 354.20, Found 355. UV-vis (nm): λ_max_ = 501.

*1(4-bromobutyl) pyrene* (**2**). Compound **2** was synthesized according the procedure previously reported by our research group [43]. The product was obtained as a white solid (290.0 mg, 0.9 mmol). Yield: 96%.

*4-(4-(pyren-1-yl)butoxy)benzaldehyde* (**3**). A solution of 1-(4-bromobutyl) pyrene (**2**) (413 mg, 1.23 mmol), p-hydroxybenzaldehyde (150 mg, 1.23 mmol), K_2_CO_3_ (238 mg, 1.72 mmol), and 18-crown-6 in DMF (20 mL) was prepared. The reaction mixture was heated to 110 °C with vigorous stirring for 4 h. Then, the solvent was removed under vacuum, the crude was taken in CH_2_Cl_2_ and washed with water. The organic phase was dried over MgSO_4_ and concentrated at reduced pressure. Finally, the product was purified by column chromatography (hexanes: ethyl acetate 6:4) in order to obtain the desired compound (**3**) as a white solid (402 mg, 1.06 mmol). Yield 86%. ^1^H NMR (CDCl_3_, 400 MHz): 9.86 (s,1H, H^28^), 8.29–7.87 (m, 9H, pyrene), 7.78 (d, 2H, H^22,24^, *J* = 8.8Hz), 6.95 (d, 2H, H^21,25^, *J =* 8.8 Hz), 4.08 (t, 2H, H^20^, *J* = 6.2 Hz), 3.44 (t, 2H, H^1^, *J* = 7.5 Hz), 2.10–1.97 (m, 4H, H^18,19^). ^13^C NMR (CDCl_3_, 100 MHz): 190.8 (C^28^), 164.1 (C^26^), 136.2 (C^23^), 131.9, 131.5, 130.9, 129.9, 129.8, 128.7, 127.5, 127.3, 127.3, 126.7 125.9, 125.1, 125.03, 124.95, 124.8, 124.8, 123.3 (16C pyrene, C^22^,C^24^), 114.7 (C^21^,C^25^), 68.1 (C^20^), 33.1 (C^1^), 29.0 (C^18^), 28.1 (C^19^). ESI: *m/z* calculated for C_27_H_22_O_2_ 378.48, Found 378.16. UV-vis (nm): λ_max_ = 344.

*5,5-difluoro-1,3,7,9-tetramethyl-10-(4-(4-(pyren-1-yl)butoxy)phenyl)-5H-dipyrrolo[1,2-c:2′,1′-f][1,3,2]diazaborinin-4-ium-5-uide* (**4**). 2,4-dimetylpyrrole (36 mg, 0.38 mmol) was reacted with zero generation dendron (**3**) (71 mg, 0.19 mmol) in presence of trifluoroacetic acid (10 μL) in anhydrous CH_2_Cl_2_ (50 mL) with vigorous stirring. After 4 h, oxidation was carried out through the addition of DDQ (43 mg, 0.19 mmol). The reaction mixture was stirred for 45 min and afterwards Et_3_N (1.5 mL) and BF_3_O(C_2_C_5_)_2_ (1.5 mL) were added. Then, it was stirred for 15 more minutes in order to obtain the final compound. The mixture was concentrated under vacuum, the crude product was placed in CH_2_Cl_2_ and washed with water. The organic phase was dried over MgSO_4_ and concentrated at reduced pressure. Finally, the product was purified by column chromatography (hexanes:ethyl acetate ratio: 6:4) in order to obtain the desired compound (**4**) as a red solid (43 mg, 0.07 mmol). Yield 38%. ^1^H NMR (CDCl_3_, 400 MHz): 8.23–7.81 (m, 9H pyrene), 7.14 (d, *J* = 8.6 Hz, 2H, H^19,23^), 6.99 (d, *J* = 8.6 Hz, 2H, H^20,22^), 5.96 (s, 2H, H^4,8^), 4.07 (t, *J* = 6.1 Hz, 2H, H^26^), 3.45 (t, *J* = 7.5 Hz, 2H, H^29^), 2.55 (s, 6H, H^16,17^), 2.20–1.92 (m, 4H, H^27,28^), 1.41 (s, 6H, H^15,18^). ^13^C NMR (CDCl_3_, 100 MHz): 159.6 (C^24^), 155.2 (C^1^), 143.2 (C^21^), 141.9 (C^3^,C^9^), 136.4 (1C pyrene), 131.9 (1C pyrene), 131.5 (1C pyrene), 130.9 (C^2^,C^10^), 129.9 (1C pyrene), 129.2 (1C pyrene, C^20^,C^22^), 128.7 (C^5^,C^7^), 127.5, 127.3, 127.0, 126.7, 125.9, 125.2, 125.04, 124.96, 124.83, 124.75, 123.3 (11C pyrene), 121.1 (C^4^,C^8^), 115.1 (C^19^,C^23^), 67.9 (C^26^), 33.2 (C^29^), 29.3 (C^27^), 28.3 (C^28^), 14.6 (C^15^,C^16^,C^17^,C^18^). MALDI-TOF: *m/z* calculated for C_39_H_35_BF_2_N_2_O 596.51. Found 597.16. UV-vis (nm): λ_max_ = 344, 501.

*1,1′-(((5-(bromomethyl)-1,3-phenylene)bis(oxy))bis(butane-4,1-diyl))dipyrene* (**5**). Compound **5** was synthesized according to the procedure previously reported by us [43]. This product was obtained as a white solid (144 mg, 0.2 mmol). Yield: 75%.

*4-((3,5-bis(4-(pyren-1-yl)butoxy)benzyl)oxy)benzaldehyde* (**6**). A solution of compound **5** (186 mg, 0.26 mmol), p-hydroxybenzaldehyde (32 mg, 0.26 mmol), K_2_CO_3_ (50 mg, 0.36 mmol), and 18-crown-6 in DMF (15 mL) was prepared. The reaction mixture was heated to 110 °C with vigorous stirring for 4 h. Then, the solvent was removed under vacuum, the crude product was dissolved in CH_2_Cl_2_ and washed with water. The organic phase was dried over MgSO_4_ and concentrated at reduced pressure. Finally, the purification was carried out by column chromatography (hexanes:ethyl acetate; 6:4) in order to obtain the desired compound (**6**) as a white solid (156 mg, 0.21 mmol). Yield 79%. ^1^H NMR (CDCl_3_, 400 MHz): 9.85 (s, 1H, H^7^), 8.28–7.86 (m, 18H, pyrene), 7.80 (d, 2H, H^1,5^, *J* = 8.8 Hz), 7.03 (d, 2H, H^2,4^, *J* = 8.8 Hz), 6.52 (d, 2H, H^12,14^, *J* = 2.2 Hz), 6.40 (t, 1H, H^16^, *J* = 2.3 Hz), 5.01 (s, 2H, H^17^), 3.98 (t, 4H, H^19,23^ *J* = 6.2 Hz), 3.41 (t, 4H, H^22,26^, *J* = 7.6 Hz), 2.06–1.92 (m, 8H, H^20,21,24,25^). ^13^C NMR (CDCl_3_, 100 MHz): 190.8 (C^7^), 163.7 (C^6^), 160.5 (C^11^,C^15^), 138.2 (C^16^), 136.5 (C^2^,C^4^), 132.0, 131.4, 130.9, 130.1, 129.9, 128.6, 127.5, 127.3, 126.6, 125.8, 125.1, 125.0, 124.9, 124.8, 124.7, 123.4 (32C of two pyrenes), 115.2 (C^12^,C^14^), 105.8 (C^1^,C^5^), 70.2 (C^17^), 67.8 (C^19^,C^23^), 33.1 (C^22^,C^26^), 29.2 (C^24^,C^20^), 28.2 (C^25^,C^21^). ESI: *m/z* calculated for C_54_H_44_O_4_ 756.32. Found [M + Na]^+^ 779.31. UV-vis (nm): λ_max_ = 344.

*10-(4-((3,5-bis(4-(pyren-1-yl)butoxy)benzyl)oxy)phenyl)-5,5-difluoro-1,3,7,9-tetramethyl-5H-dipyrrolo[1,2-c:2′,1′-f][1,3,2]diazaborinin-4-ium-5-uide* (**7**). 2,4,-dimethylpyrrole (19 mg, 0.2 mmol) was reacted with generation 1 dendron (**6**) (76 mg, 0.1 mmol) in the presence of trifluoroacetic acid (10 μL) in anhydrous CH_2_Cl_2_ (50 mL) with vigorous stirring. After 4 h, an oxidation was carried out through the addition of DDQ (23 mg, 0.1 mmol). The reaction mixture was stirred for 45 min and then Et_3_N (1.00 mL) and BF_3_O(Et)_2_ (1.00 mL) were added. Afterward, it was stirred for 15 more min in order to obtain the final compound. The mixture was concentrated under vacuum, the crude product was dissolved in CH_2_Cl_2_ and washed with water. The organic phase was dried over MgSO_4_ and concentrated at reduced pressure. Finally, the product was purified by column chromatography (hexanes:ethyl acetate; 6:4) in order to obtain the desired compound (**7**) as a red solid (50 mg, 0.06 mmol). Yield 47%. ^1^H NMR (CDCl_3_, 400 MHz): 8.28–7.88 (m,18H pyrene), 7.11 (d, *J* = 8.6 Hz, 2H H^15,17^), 7.02 (d, *J* = 8.7, 2H, H^14,18^), 6.55 (d, *J* = 2.2, 2H, H^28,30^), 6.41 (s, 1H, H^32^), 5.89 (s, 2H, H^8,11^), 5.01 (s, 2H, H^33^), 3.99 (t, *J* = 6.2, 4H, H^35,39^), 3.42 (t, *J* = 7.6, 4H, H^38,42^), 2.53 (s, 6H, H^22,24^), 2.11–1.87 (m, 8H, H^36,37,40,41^), 1.37 (s, 6H, H^21,23^). ^13^C NMR (CDCl_3_, 100 MHz): 160.0 (C^16^), 158.7 (C^27^,C^31^), 154.7 (C^6^), 142.6 (C^9^,C^10^,C^13^), 138.2 (2C pyrene), 136.0 (C^23^,C^29^), 130.9 (C^1^,C^15^), 130.4, 129.4, 128.7, 128.1, 127.0, 126.8, 126.1, 125.3, 124.6, 124.5, 124.4, 124.3, 124.2, 122.9 (30C pyrene, C^7^,C^12^), 120.6 (C^11^,C^8^), 115.1 (C^15^,C^17^), 105.2 (C^28^,C^30^), 100.4 (C^32^), 69.5 (C^33^), 67.3 (C^35^,C^39^), 32.7 (C^38^,C^42^), 28.7 (C^36^,C^40^), 27.8 (C^37^,C^41^), 14.0 (C^21^,C^22^,C^23^,C^24^). MALDI-TOF: *m/z* calculated for C_66_H_57_BF_2_N_2_O_3_ 974.98 Found: 974.4. UV-vis (nm): λ_max_ = 344, 501.

## 4. Conclusions

A new series of dyads bearing pyrene-bodipy units as the donor-acceptor pair was synthesized and characterized. We demonstrated that both chromophores do not interact in the ground state. The spectroscopic studies proved that a FRET phenomenon occurs in dyads **4** and **7**. FRET efficiency showed high values, close to 100%, for both compounds. Naked eye observation of the compounds in solution showed the typical bodipy emission (green), thereby confirming the results of this study. The emission quantum yields of this new series were not significantly affected by the presence of oxygen. For future perspective, these compounds can be applied for Organic Light Emitting Diodes (OLED’s) or chemosensor devices.

## Figures and Tables

**Table 1:** Absorption coefficients, quantum yields, and fluorescence resonance energy transfer (FRET) efficiencies for the pyrene-labelled aldehyde precursors **3** and **6** and the pyrene-labeled bodipy compounds **1**, **4**, and **7**.

Compound	λ_max_ Absorption (nm)/ε(M^−1^cm^−1^) ^a^	λ_max_ Emission (nm)	Φpyrene ^b^ λ_ex_ = 344	Φbodipy ^c^ λ_ex_ = 344	Φbodipy ^c^ λ_ex_ = 475	E_FRET_^d^
**3**	344/54,000	375	0.30 ± 0.03	-	-	-
**6**	344/101,000	375, 480	0.39 ± 0.02	-	-	-
**1**	501/87,000	509	-	0.32 ± 0.01	0.71 ± 0.02	-
**4**	344/47,000 501/79,000	509	0.0085 ± 0.0004	0.48 ± 0.02	0.65 ± 0.01	0.98
**7**	344/87,000 501/70,000	509	0.0097 ± 0.0003	0.49 ± 0.01	0.71 ± 0.01	0.99

^a^ Values of the absorption coefficients calculated at 344 and 501 nm for the pyrene and bodipy part, respectively. ^b^ All the Φpyrene was calculated using quinine in H_2_SO_4_ 1 M solution as the reference (0.54) [40,41]. ^c^ All the Φbodipy were calculated using fluorescein in NaOH 0.1 M solution as the reference (0.92) [42]. ^d^ FRET efficiencies were calculated with the subsequent equation: E_FRET_ = 1 − I_(Py+Bodipy)_/I_(Py)_, where I_(Py+Bodipy)_ is the integration of the residual emission of pyrene when linked to the bodipy acceptor and I_(Py)_ is the integration of the emission of pyrene in the absence of the bodipy acceptor [41].

## Supplementary Materials

The following are available online.
