# Supplementary material for: Design of Novel Pyrene-Bodipy Dyads: Synthesis, Characterization, Optical Properties, and FRET Studies
Source: Molecules. 2018 Sep 7;23(9):2289. doi: 10.3390/molecules23092289 (PMC6225113; doi:10.3390/molecules23092289)
Supplement: Supplementary file 1 [file molecules-23-02289-s001.pdf]

**Supporting information**

**For:**

# "Design of Novel Pyrene-Bodipy Dyads: Synthesis, Characterization, Optical Properties and FRET studies"

Pasquale Porcu, Mireille Vonlanthen, Israel González-Méndez, Andrea Ruiu and Ernesto Rivera*

1 Instituto de Investigaciones en Materiales, Universidad Nacional Autónoma de México.

**Table of contents:**

1. $^{1}$HNMR, $^{13}$CNMR, mass spectra of compounds **1**,**3**,**4**,**6**,**7** **S3-S19**
2. Absorption/Emission spectra of compounds **1,3**,**4** **S20-S22**
3. Absorption/Excitation spectra of compounds **6**,**7** **S23-S24**

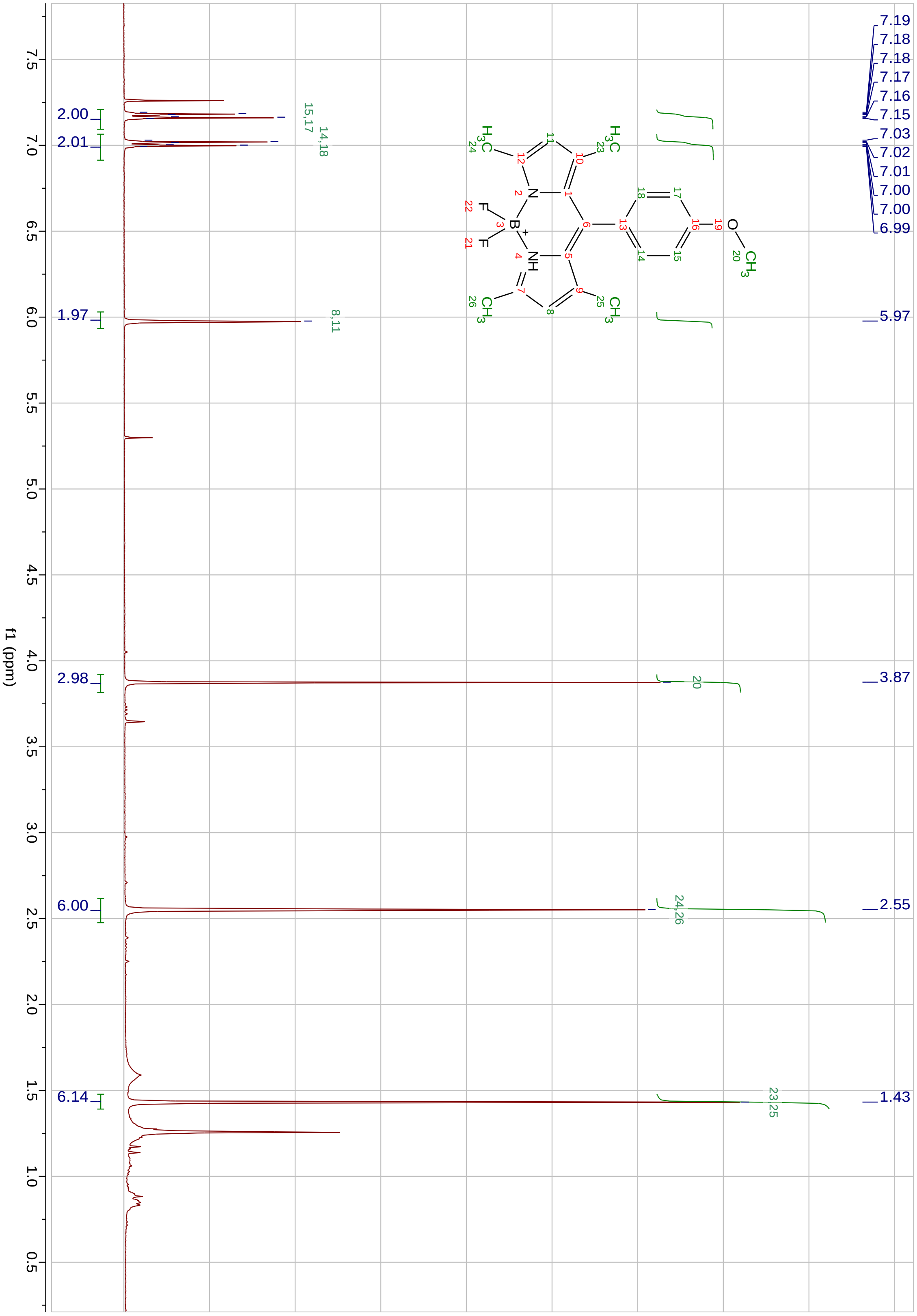
f1 (ppm)
7.19
7.18
7.18
7.17
7.16
7.15
7.03
7.02
7.01
7.00
7.00
6.99
5.97
3.87
2.55
1.43
2.00
2.01
1.97
2.98
6.00
6.14
15,17
14,18
8,11
20
24,26
23,25
H3C
CH3
O
N
NH
B
F
F

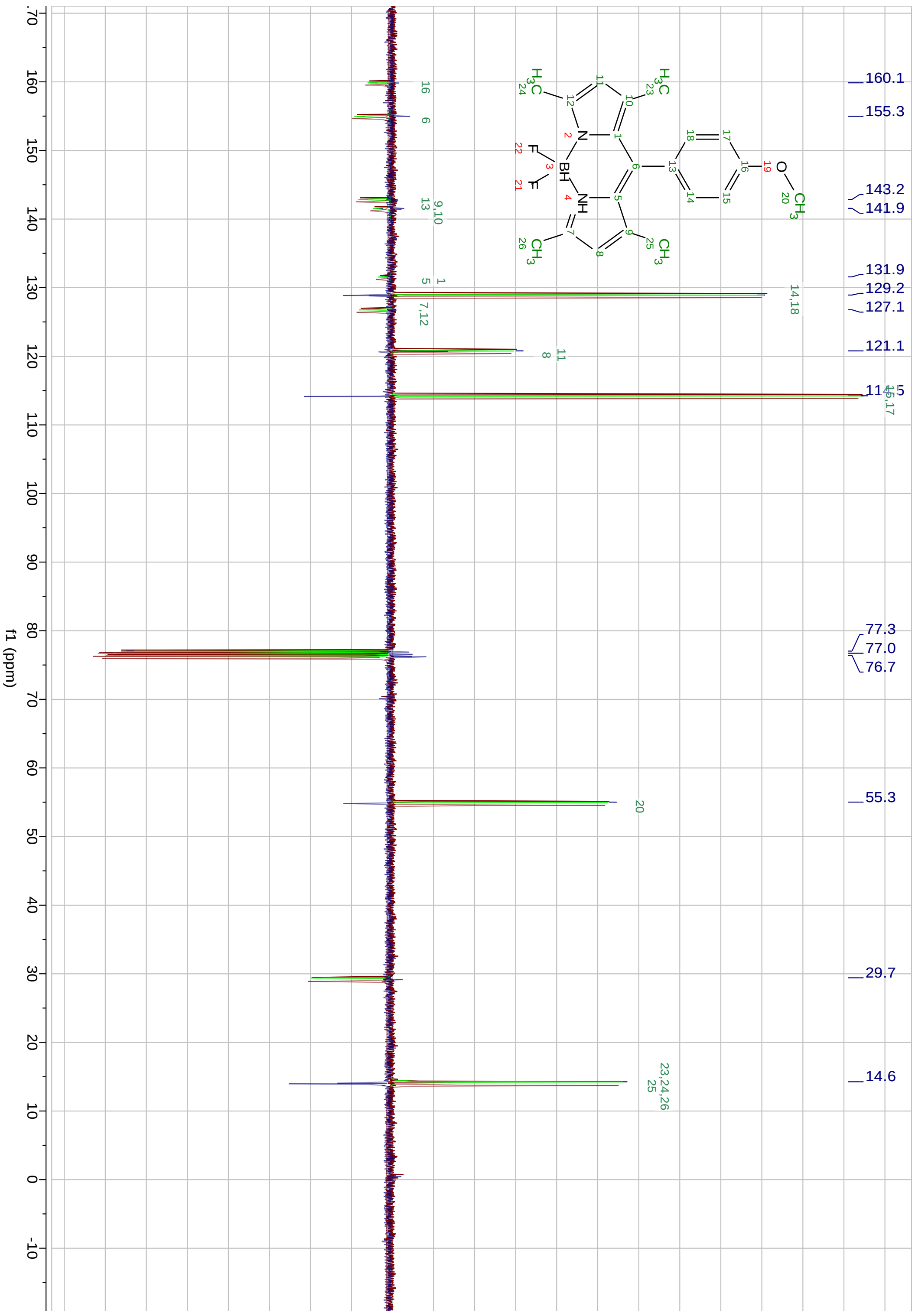

f1 (ppm)
160.1
155.3
143.2
141.9
131.9
129.2
127.1
121.1
114.5
77.3
77.0
76.7
55.3
29.7
14.6

Experiment Date/Time: 10/27/2016 12:22:30 PM
Creation Parameters: Average(MS[1] Time:0.81..0.84)

Acq. Data Name: 331 STA-2400 BODBENZOCH3
Ionization Mode:DART + :

Operator Name: Carmen Gacia-Javier Perez: AccuTOF
Instrument: JEOL The AccuTOF: JMS-T100LC

Dr Pasquale Porcu/ Operador:Carmen Garcia-Javier Perez Instituto de Quimica-UNAM:

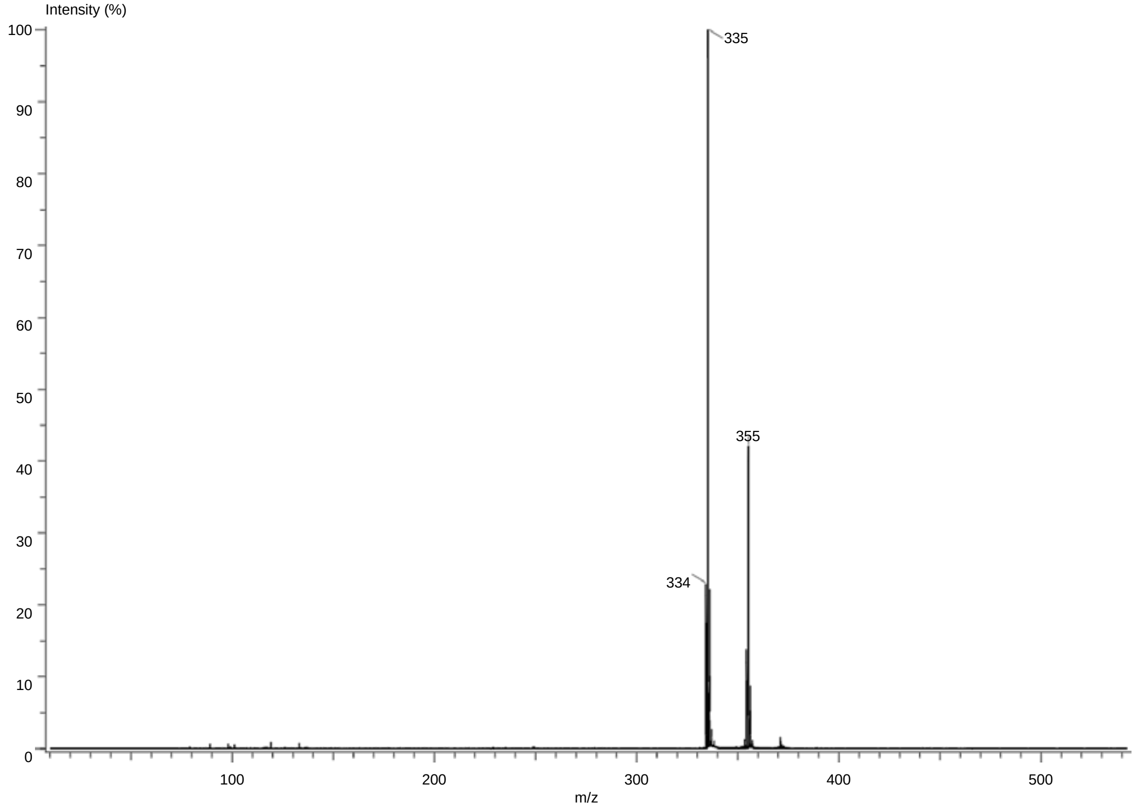

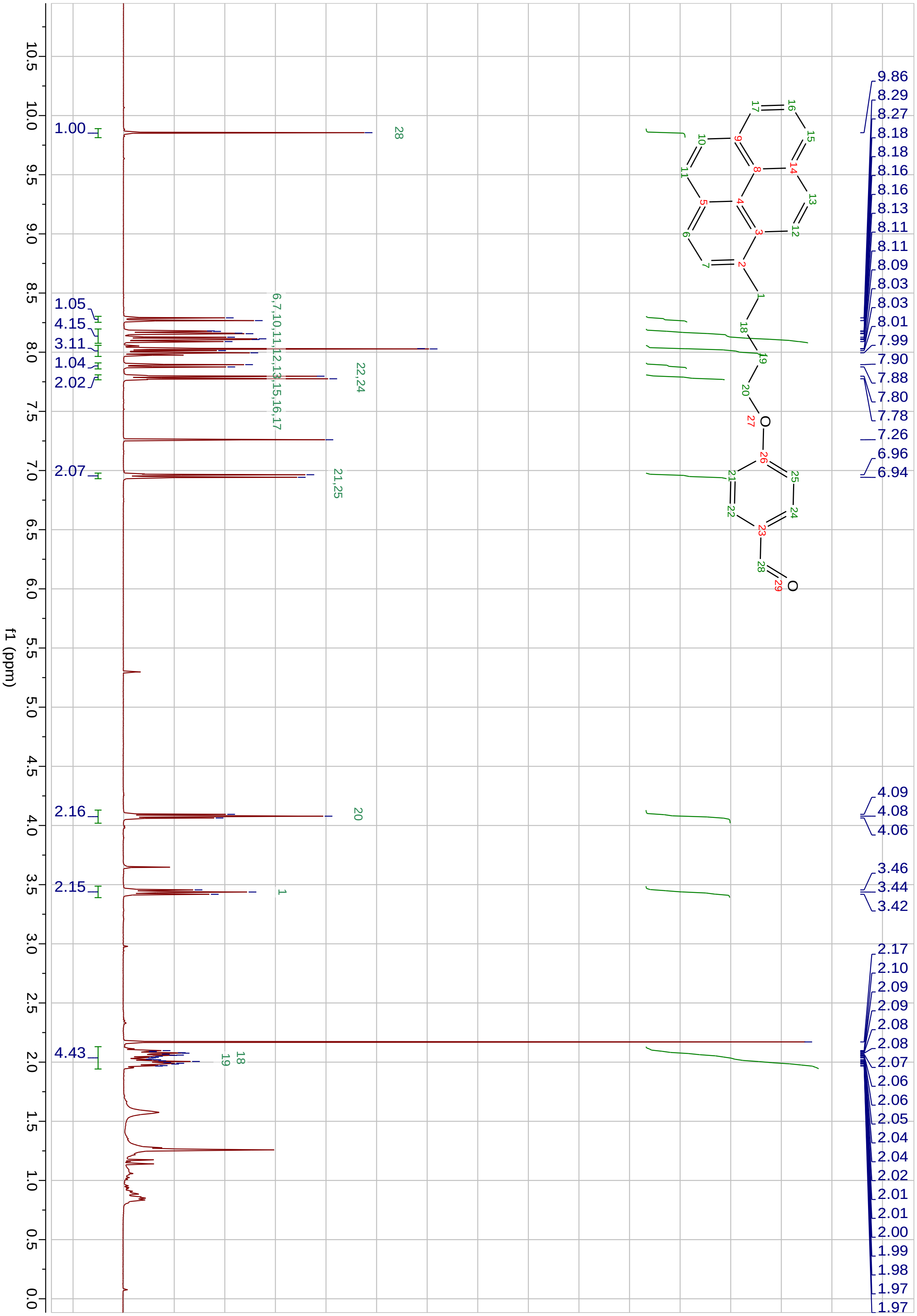

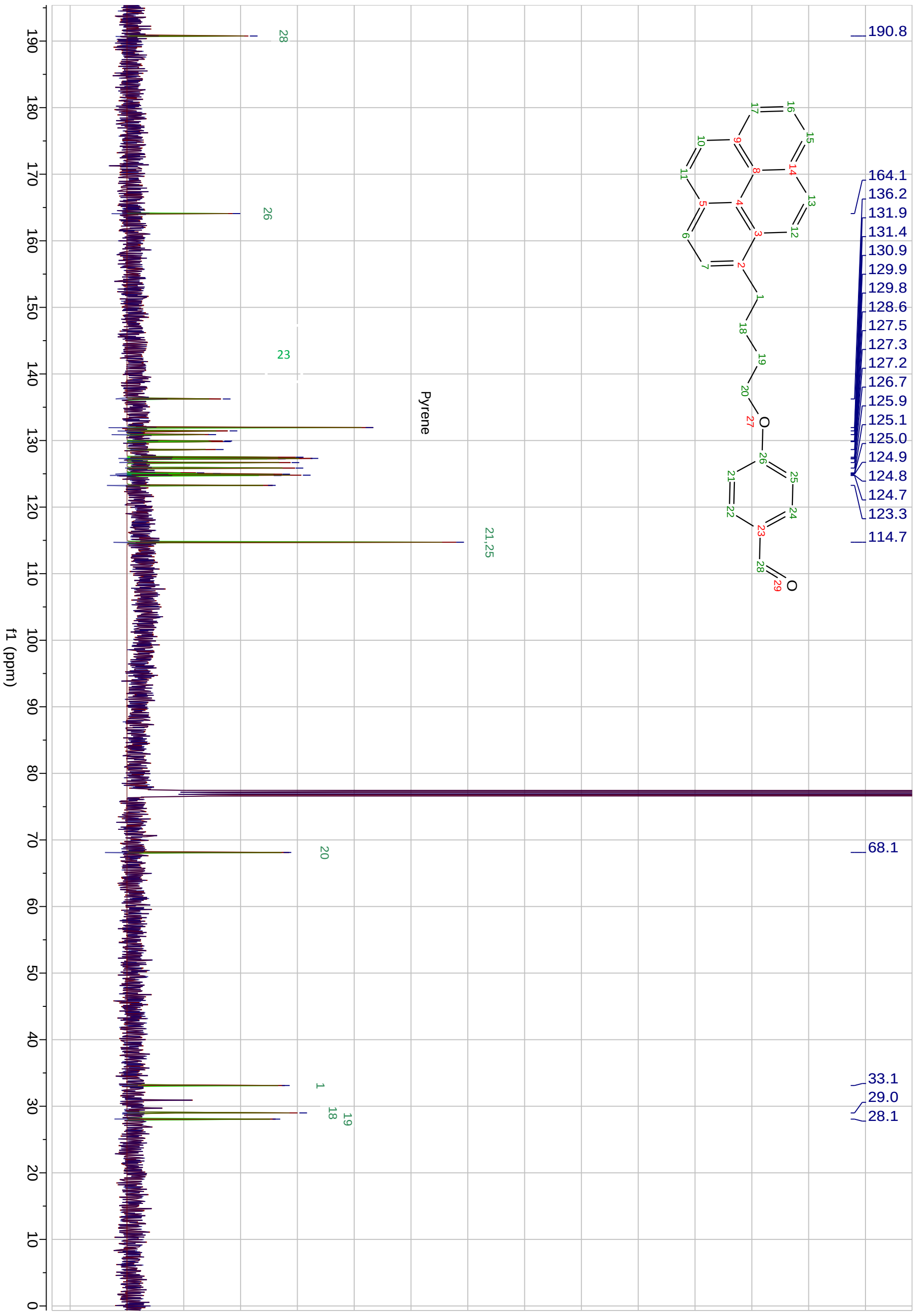
f1 (ppm)
Pyrene
190.8
164.1
136.2
131.9
131.4
130.9
129.9
129.8
128.6
127.5
127.3
127.2
126.7
125.9
125.1
125.0
124.9
124.8
124.7
123.3
114.7
68.1
33.1
29.0
28.1

# Qualitative Analysis Report

| | | | |
|---|---|---|---|
| **Data Filename** | Dr RIBERA2 .d | **Sample Name** | Py1G0HO |
| **Sample Type** | Sample | **Position** | P1-C2 |
| **Instrument Name** | LC QTOF_LANCIC | **User Name** | |
| **Acq Method** | iny dir.m | **Acquired Time** | 3/22/2018 1:20:37 PM |
| **IRM Calibration Status** | Success | **DA Method** | carmen1.m |
| **Comment** | | | |

| | | | |
|---|---|---|---|
| **Sample Group** | | **Info.** | |
| **Stream Name** | LC 1 | **Acquisition SW Version** | 6200 series TOF/6500 series Q-TOF B.06.01 (B6172 SP1) |

## User Spectra

| **Spectrum Source** | **Fragmentor Voltage** | **Collision Energy** | **Ionization Mode** |
|---|---|---|---|
| Peak (1) in "+ TIC Scan - Dr RIBERA2 .d" | 240 | 50 | ESI |

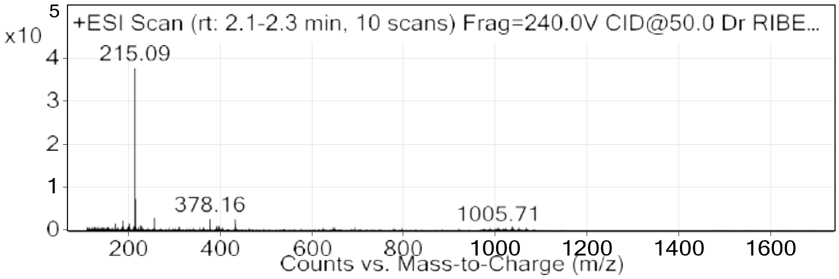


**Peak List**

| *m/z* | z | Abund |
|---|---|---|
| 172.86 | | 17778.46 |
| 189.07 | 1 | 24365.61 |
| 202.08 | 1 | 18246.88 |
| 215.09 | 1 | 382522.03 |
| 216.09 | 1 | 73669.32 |
| 228.09 | | 13284.12 |
| 257.13 | 1 | 30722.55 |
| 378.16 | 1 | 27877.57 |
| 433.09 | 1 | 27795.62 |
| 434.09 | 1 | 14255.7 |

--- End Of Report ---

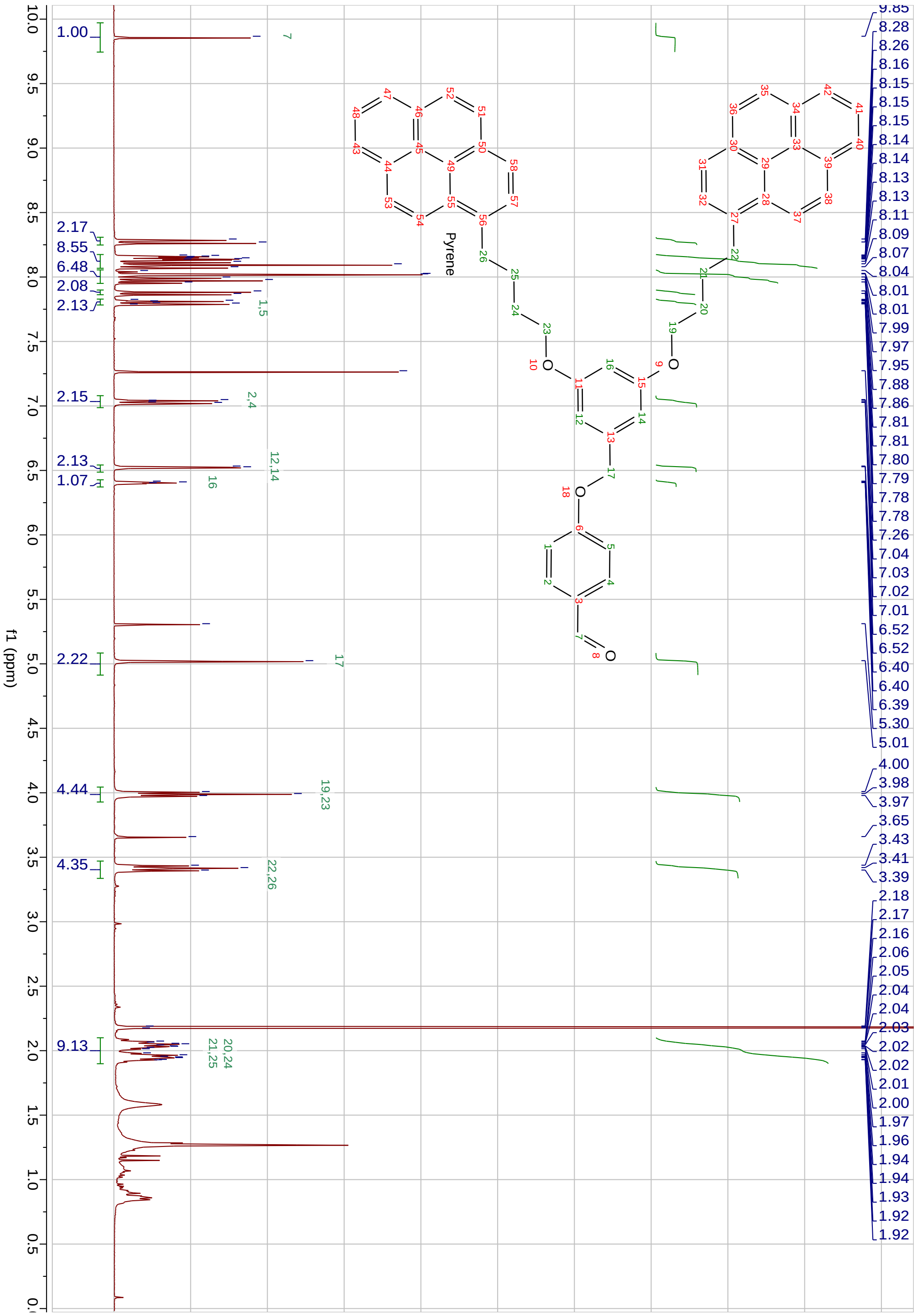
Pyrene
f1 (ppm)
1.00
2.17
8.55
6.48
2.08
2.13
2.15
2.13
1.07
2.22
4.44
4.35
9.13
9.85
8.28
8.26
8.16
8.15
8.15
8.15
8.14
8.14
8.13
8.13
8.11
8.09
8.07
8.04
8.01
8.01
7.99
7.97
7.95
7.88
7.86
7.81
7.81
7.80
7.79
7.78
7.78
7.26
7.04
7.03
7.02
7.01
6.52
6.52
6.40
6.40
6.39
5.30
5.01
4.00
3.98
3.97
3.65
3.43
3.41
3.39
2.18
2.17
2.16
2.06
2.05
2.04
2.04
2.03
2.02
2.02
2.01
2.00
1.97
1.96
1.94
1.94
1.93
1.92
1.92

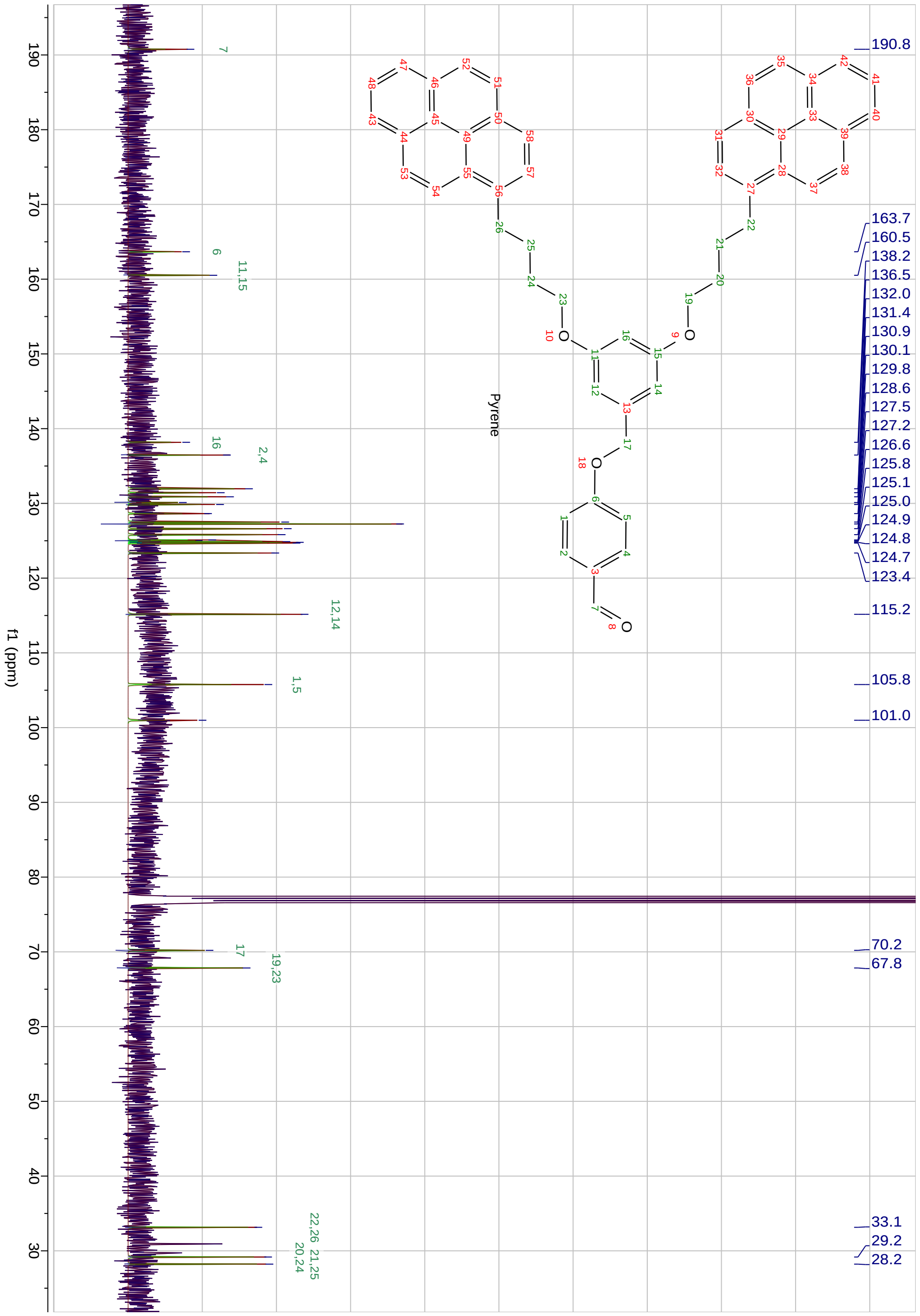

Pyrene
f1 (ppm)
190.8
163.7
160.5
138.2
136.5
132.0
131.4
130.9
130.1
129.8
128.6
127.5
127.2
126.6
125.8
125.1
125.0
124.9
124.8
124.7
123.4
115.2
105.8
101.0
70.2
67.8
33.1
29.2
28.2

# Qualitative Analysis Report

| | | | |
|---|---|---|---|
| **Data Filename** | Dr RIBERA3 .d | **Sample Name** | Py1G1HO |
| **Sample Type** | Sample | **Position** | P1-C3 |
| **Instrument Name** | LC QTOF_LANCIC | **User Name** | |
| **Acq Method** | iny dir.m | **Acquired Time** | 3/22/2018 1:27:39 PM |
| **IRM Calibration Status** | Success | **DA Method** | carmen1.m |

**Comment**

**Sample Group**

**Stream Name** LC 1

**Info.**

**Acquisition SW Version** 6200 series TOF/6500 series Q-TOF B.06.01 (B6172 SP1)

## User Spectra

**Spectrum Source**

+ TIC Scan

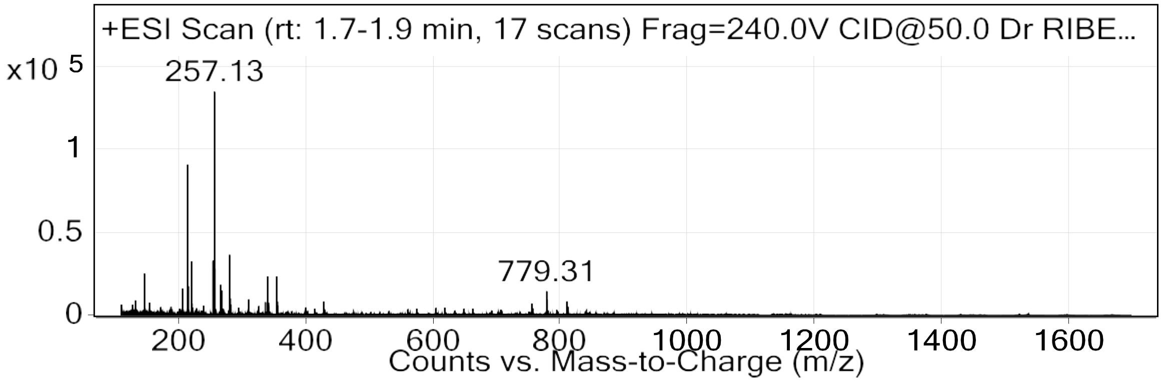


**Peak List**

| *m/z* | z | Abund |
|---|---|---|
| 147.07 | 1 | 26323.41 |
| 215.09 | 1 | 91808.67 |
| 221.08 | 1 | 33339.86 |
| 255.12 | | 33034.48 |
| 257.13 | 1 | 134774.97 |
| 258.14 | 1 | 28120.25 |
| 267 | 1 | 18441.18 |
| 281.05 | 1 | 36439.58 |
| 341.02 | 1 | 23973.43 |
| 355.07 | 1 | 23757.11 |

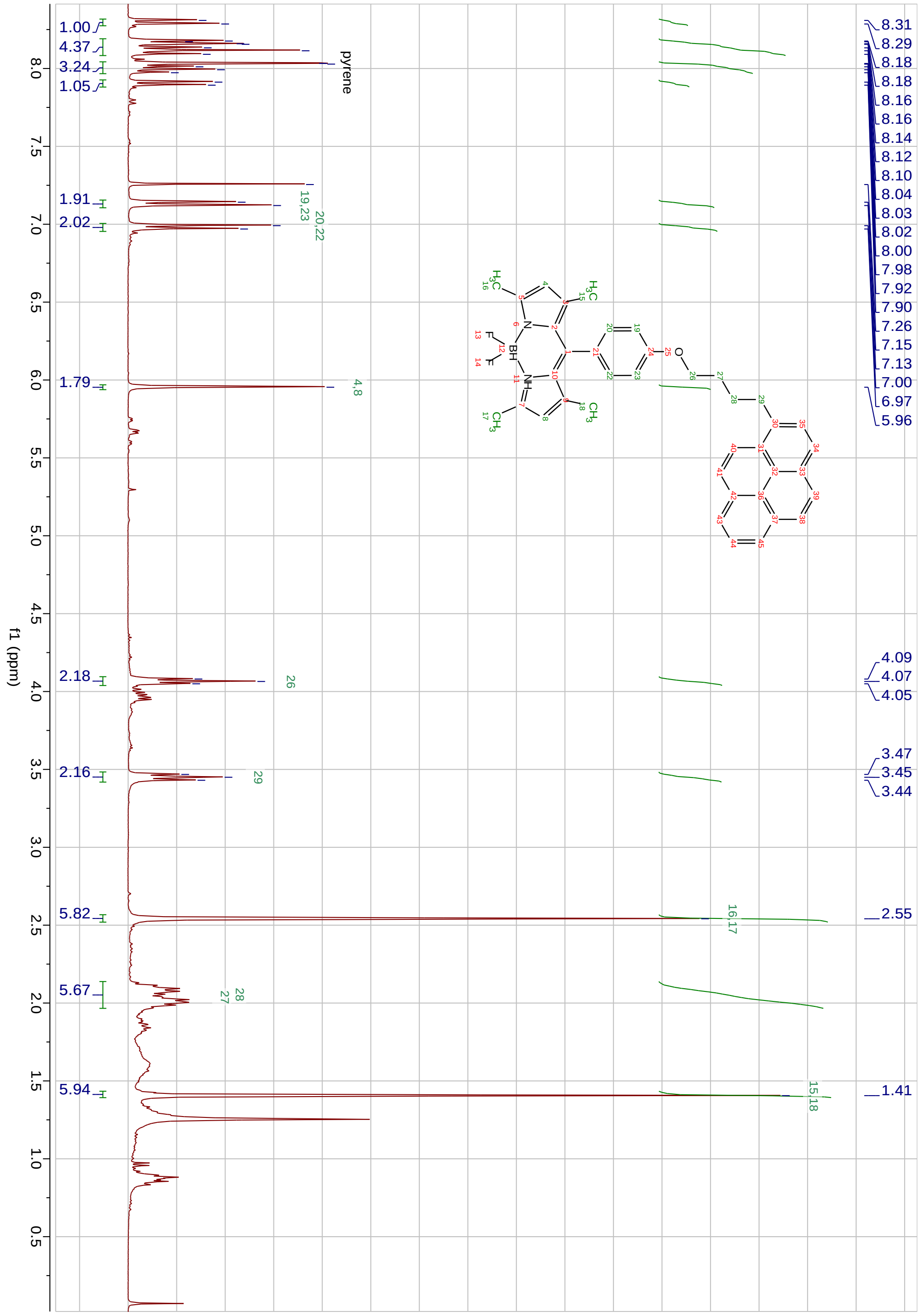

f1 (ppm)
pyrene
1.00
4.37
3.24
1.05
1.91
2.02
1.79
2.18
2.16
5.82
5.67
5.94
19,23
20,22
4,8
26
29
16,17
28
27
15,18
8.31
8.29
8.18
8.18
8.16
8.16
8.14
8.12
8.10
8.04
8.03
8.02
8.00
7.98
7.92
7.90
7.26
7.15
7.13
7.00
6.97
5.96
4.09
4.07
4.05
3.47
3.45
3.44
2.55
1.41

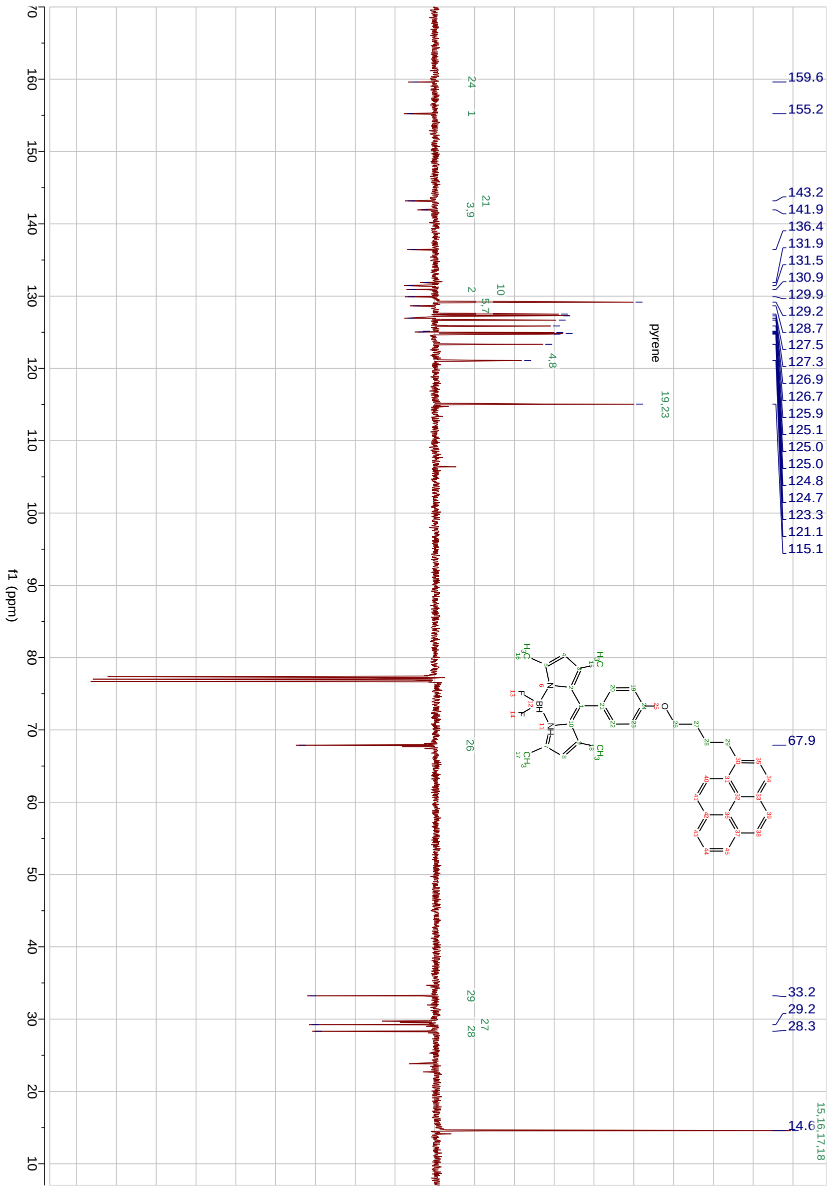
f1 (ppm)
pyrene
159.6
155.2
143.2
141.9
136.4
131.9
131.5
130.9
129.9
129.2
128.7
127.5
127.3
126.9
126.7
125.9
125.1
125.0
125.0
124.8
124.7
123.3
121.1
115.1
67.9
33.2
29.2
28.3
14.6

Registro BODPyGo 180517-ext-01 STA 2851
Matriz DIT 2/5

**Laboratorios de Servicios Analiticos**
**Instituto de Quimica UNAM**

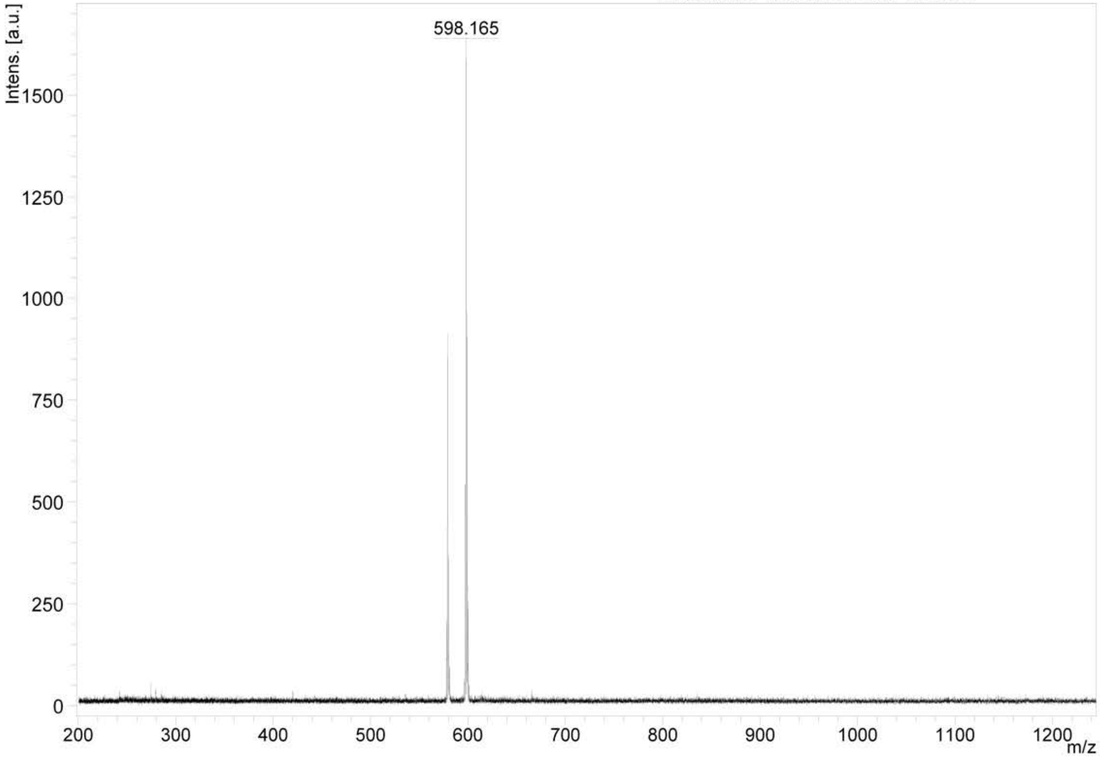


**Acquisition Parameter**

| | |
|---|---|
| *Date of acquisition* | 2018-05-24T12:23:03.671-05:00 |
| *Acquisition method name* | D:\Methods\flexControlMethods\LNM_UNAM\RP_PepMix.par |
| *Aquisition operation mode* | Reflector |
| *Voltage polarity* | POS |
| *Number of shots* | 150 |
| *Name of spectrum used for calibration* | |
| *Calibration reference list used* | |

**Instrument Info**

| | |
|---|---|
| *User* | UNAM |
| *Instrument* | FLEX-PC |
| *Instrument type* | microflex |

**D:\data\LSA\BODPyGo 180517-ext-01\0_D4\3**

Bruker Daltonics flexAnalysis printed: 5/24/2018 12:26:14 PM

Registro BODPyGo 180517-ext-01 STA 2851
Matriz DIT 2/5

**Laboratorios de Servicios Analiticos**
**Instituto de Quimica UNAM**

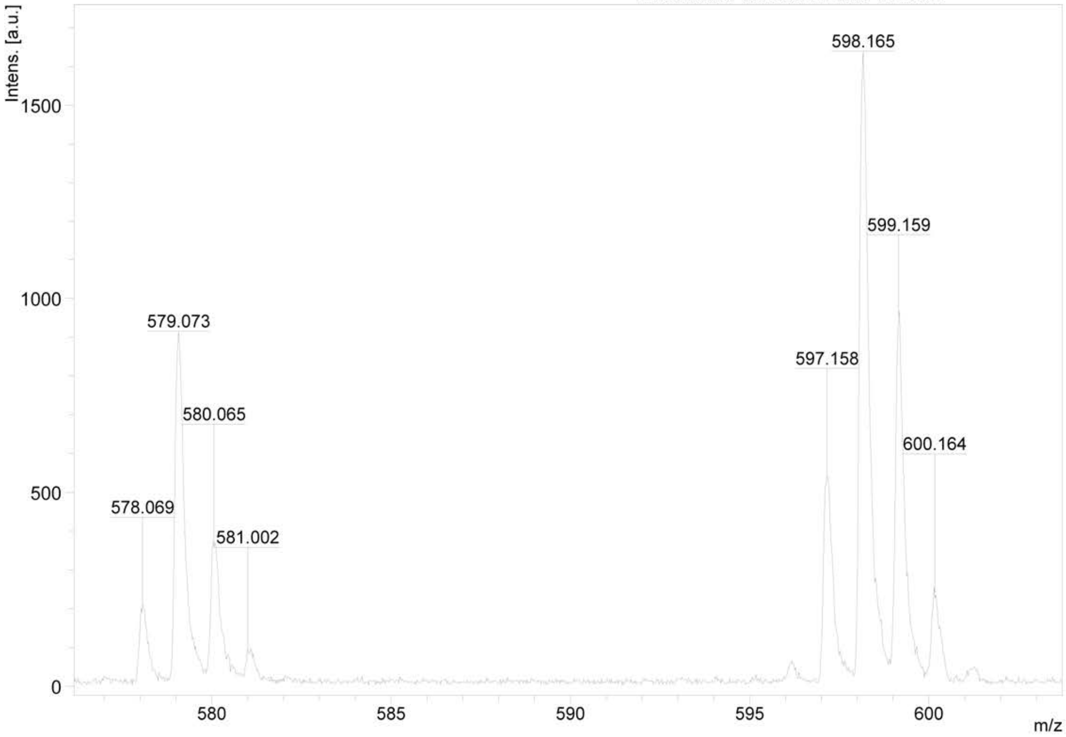


**Acquisition Parameter**

| | |
|---|---|
| *Date of acquisition* | 2018-05-24T12:23:03.671-05:00 |
| *Acquisition method name* | D:\Methods\flexControlMethods\LNM_UNAM\RP_PepMix.par |
| *Aquisition operation mode* | Reflector |
| *Voltage polarity* | POS |
| *Number of shots* | 150 |
| *Name of spectrum used for calibration* | |
| *Calibration reference list used* | |

**Instrument Info**

| | |
|---|---|
| *User* | UNAM |
| *Instrument* | FLEX-PC |
| *Instrument type* | microflex |

**D:\data\LSA\BODPyGo 180517-ext-01\0_D4\3**

Bruker Daltonics flexAnalysis printed: 5/24/2018 12:28:09 PM

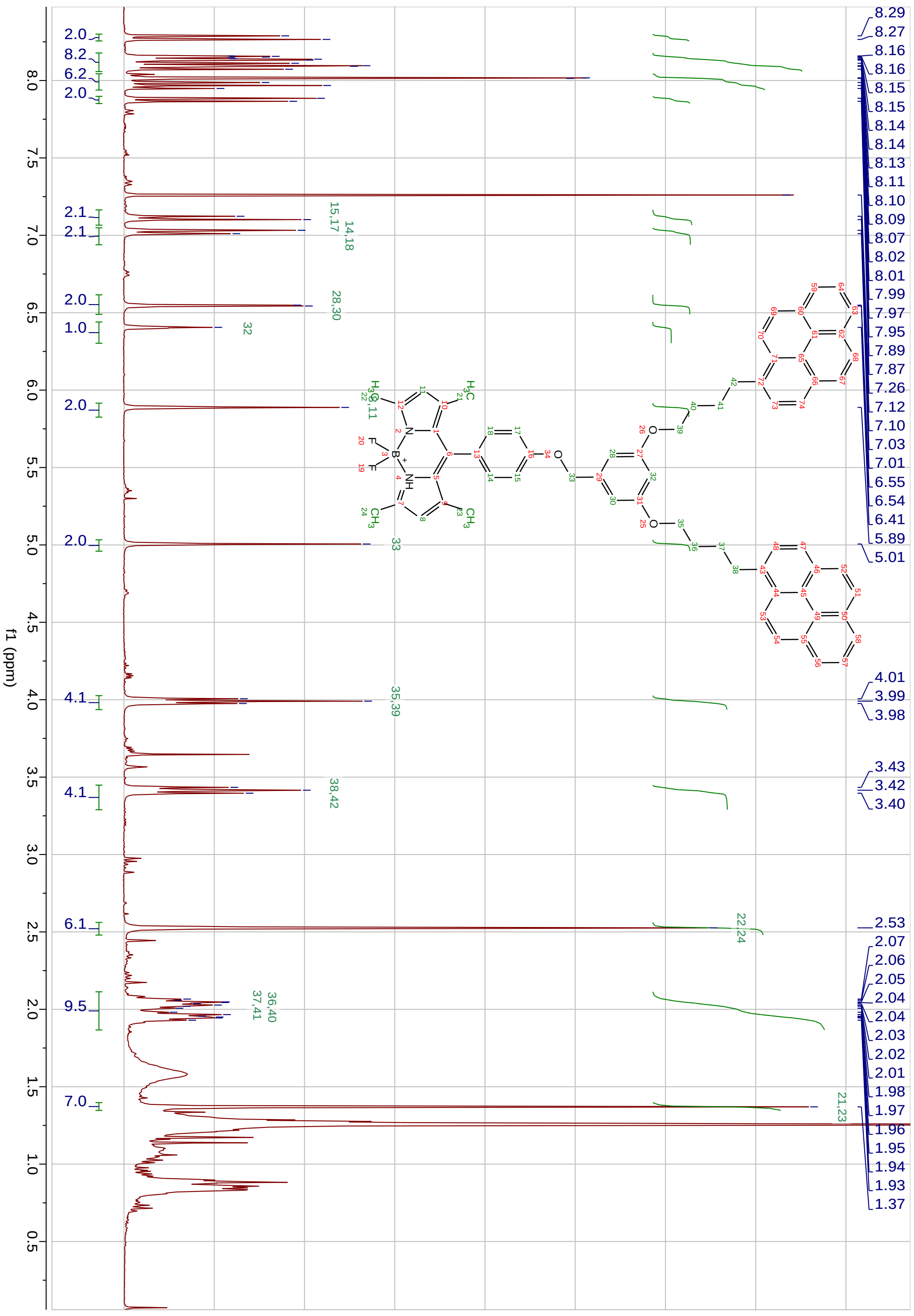

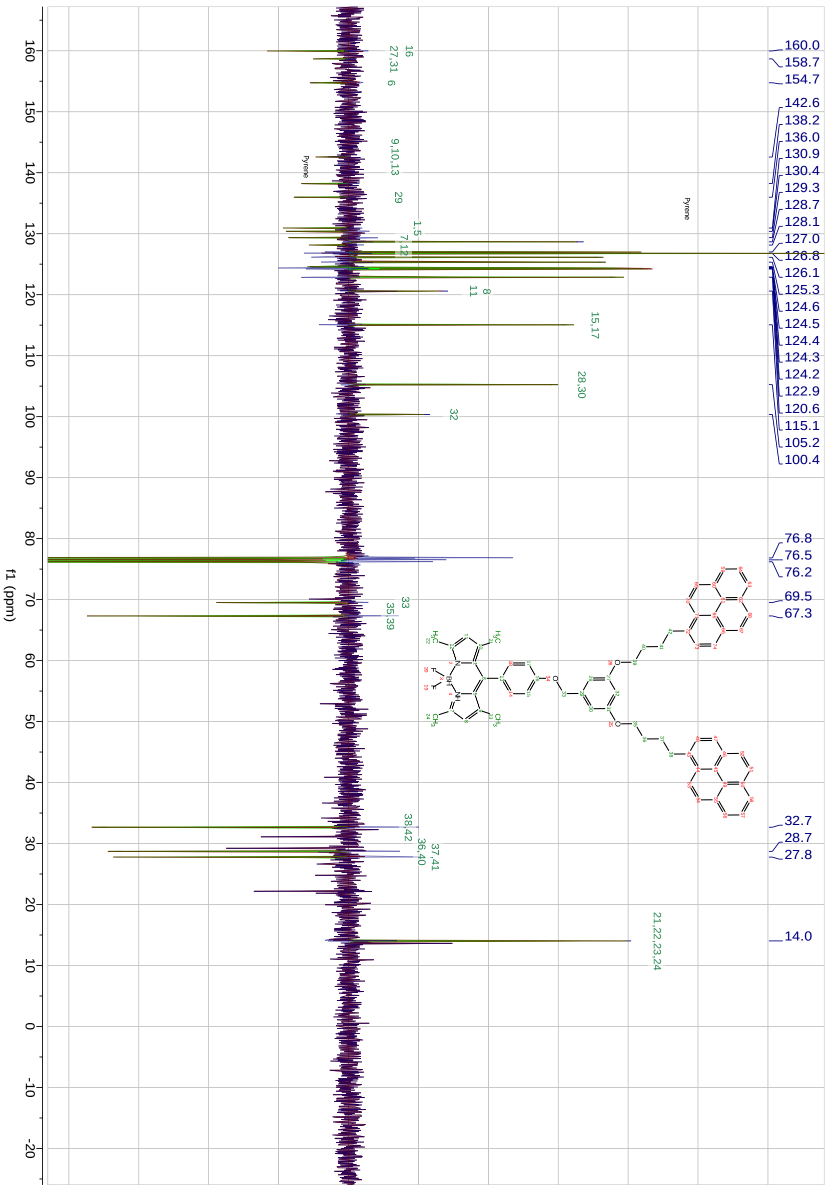

160.0
158.7
154.7
142.6
138.2
136.0
130.9
130.4
129.3
128.7
128.1
127.0
126.8
126.1
125.3
124.6
124.5
124.4
124.3
124.2
122.9
120.6
115.1
105.2
100.4
76.8
76.5
76.2
69.5
67.3
32.7
28.7
27.8
14.0
Pyrene
f1 (ppm)

Registro Py2G1BOD
Matriz DHB 2/5 180424-ext-01 STA 2824

**Laboratorios de Servicios Analiticos**
**Instituto de Quimica UNAM**

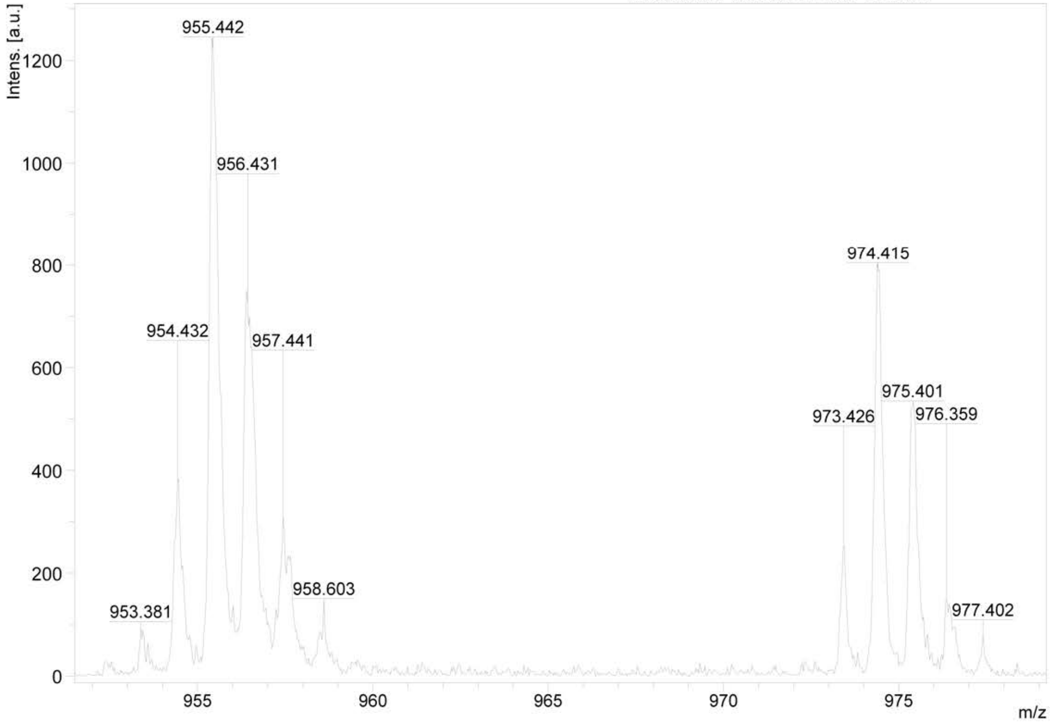


**Acquisition Parameter**

| | |
|---|---|
| *Date of acquisition* | 2018-04-25T13:27:51.250-05:00 |
| *Acquisition method name* | D:\Methods\flexControlMethods\LNM_UNAM\RP_PepMix.par |
| *Aquisition operation mode* | Reflector |
| *Voltage polarity* | POS |
| *Number of shots* | 74 |
| *Name of spectrum used for calibration* | |
| *Calibration reference list used* | |

**Instrument Info**

| | |
|---|---|
| *User* | UNAM |
| *Instrument* | FLEX-PC |
| *Instrument type* | microflex |

**D:\data\LSA\Py2G1BOD\0_D10\1**

Bruker Daltonics flexAnalysis printed: 4/25/2018 3:04:54 PM

Registro Py2G1BOD
Matriz DHB 2/5 180424-ext-01 STA 2824

**Laboratorios de Servicios Analiticos**
**Instituto de Quimica UNAM**

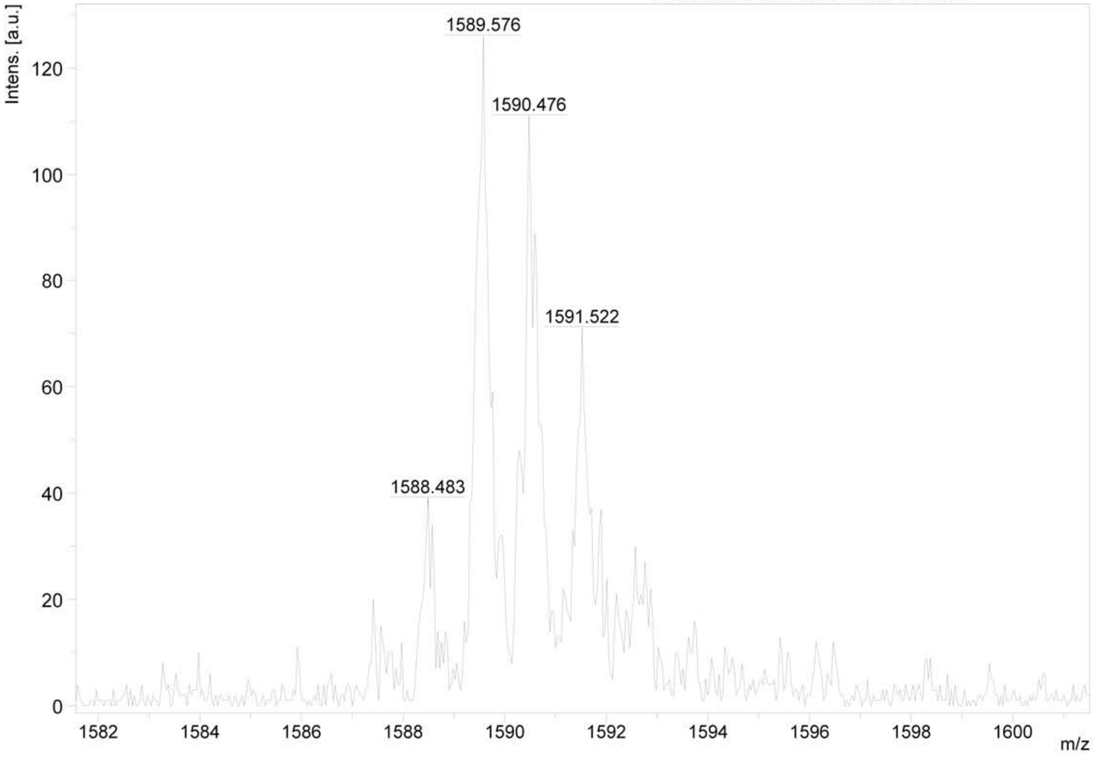


**Acquisition Parameter**

| | |
|---|---|
| *Date of acquisition* | 2018-04-25T13:27:51.250-05:00 |
| *Acquisition method name* | D:\Methods\flexControlMethods\LNM_UNAM\RP_PepMix.par |
| *Aquisition operation mode* | Reflector |
| *Voltage polarity* | POS |
| *Number of shots* | 74 |
| *Name of spectrum used for calibration* | |
| *Calibration reference list used* | |

**Instrument Info**

| | |
|---|---|
| *User* | UNAM |
| *Instrument* | FLEX-PC |
| *Instrument type* | microflex |

**D:\data\LSA\Py2G1BOD\0_D10\1**

Bruker Daltonics flexAnalysis **printed: 4/25/2018 3:06:22 PM**

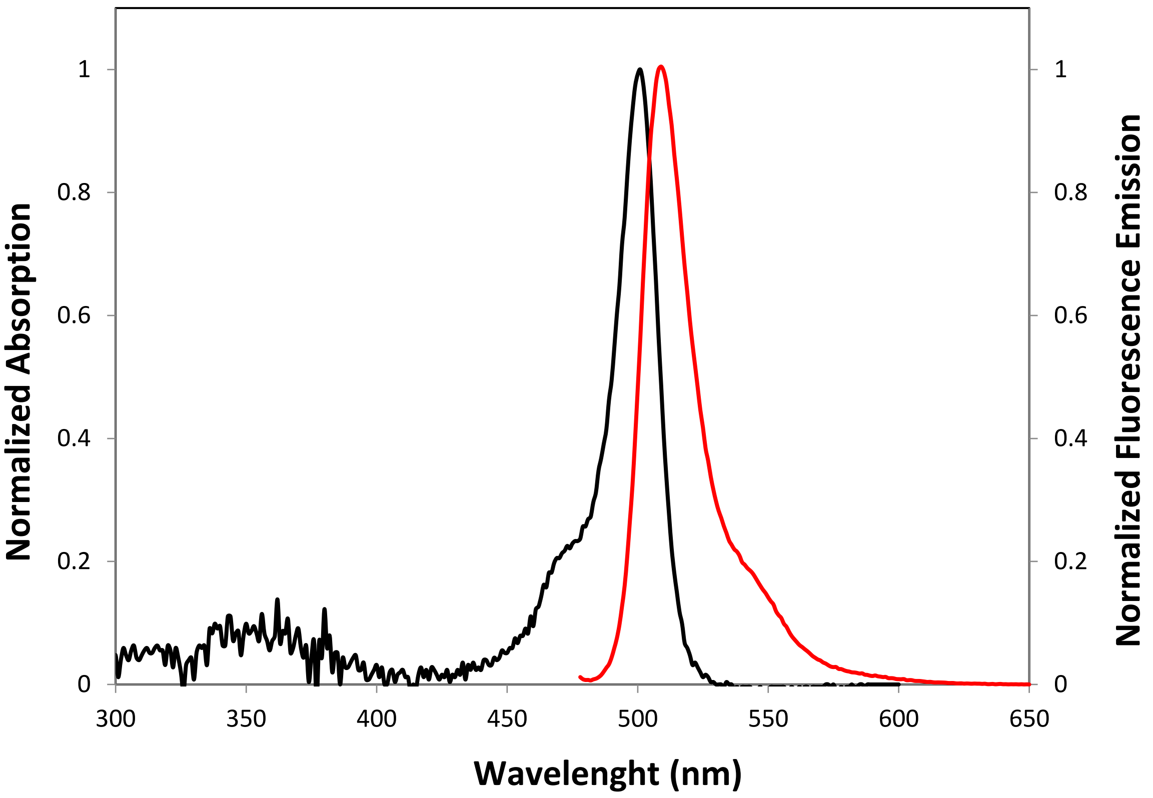


Figure S1: absorption (black line) and emission (red line) spectra of compound **1**

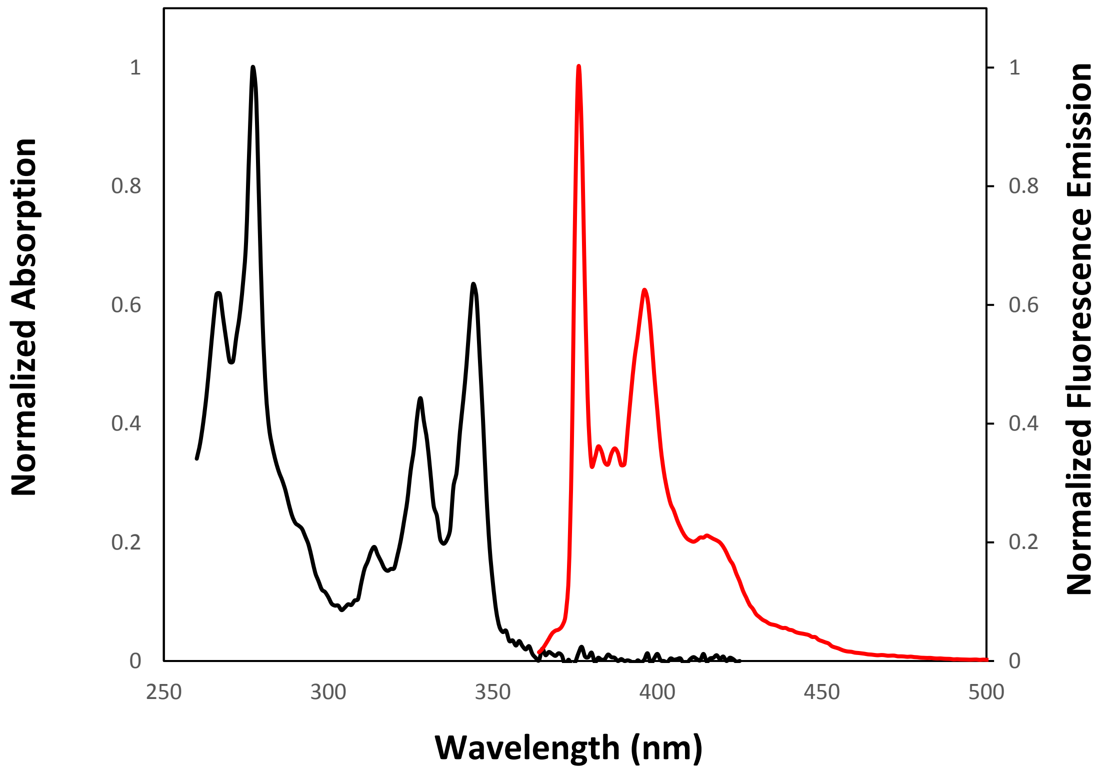


Figure S2: absorption (black line) and emission (red line) spectra of compound **3**

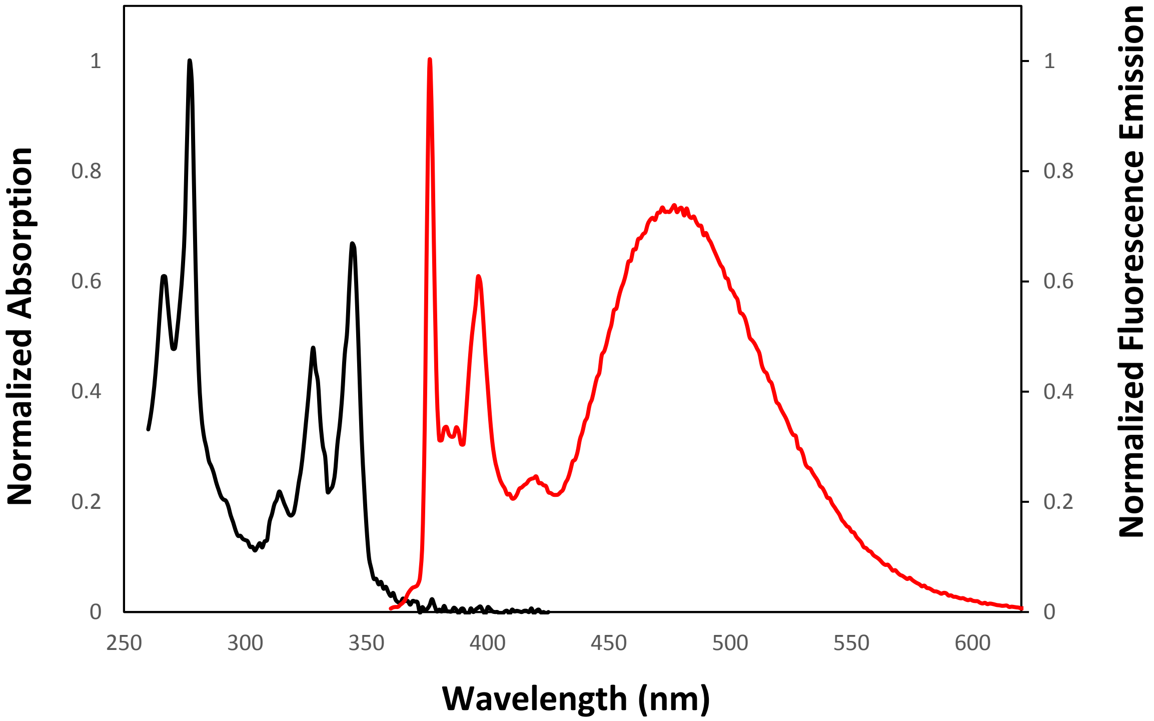


Figure S3: absorption (black line) and emission (red line) spectra of compound **6**

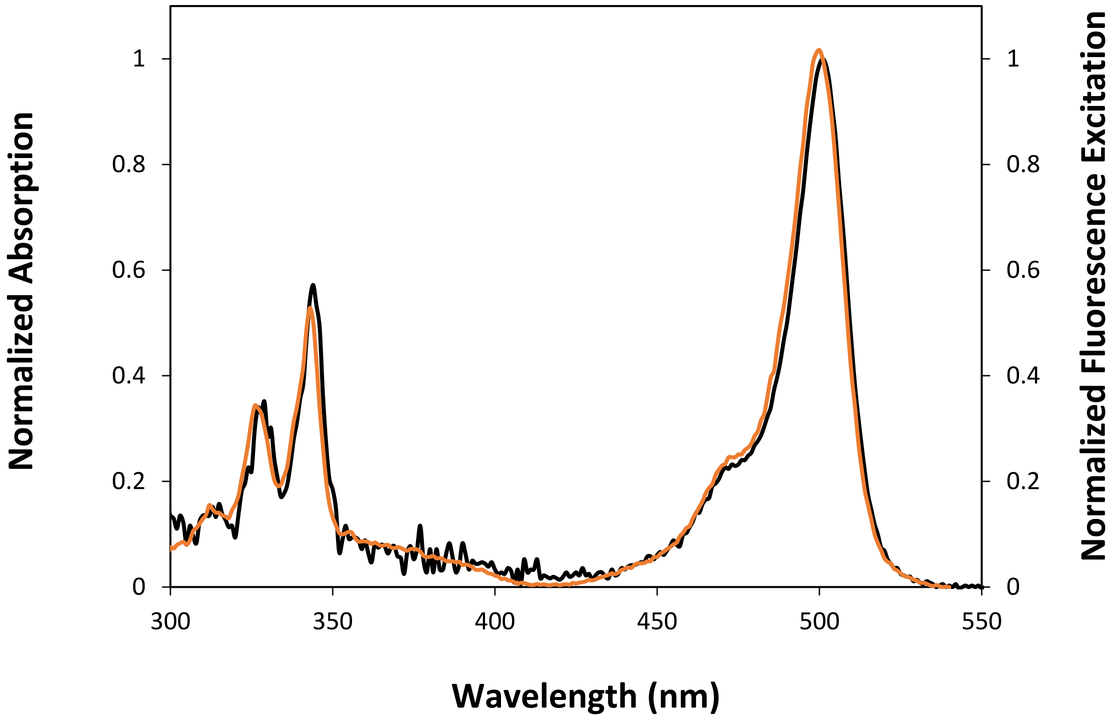


Figure S4: absorption (black line) and excitation (red line) spectra of compound **4**

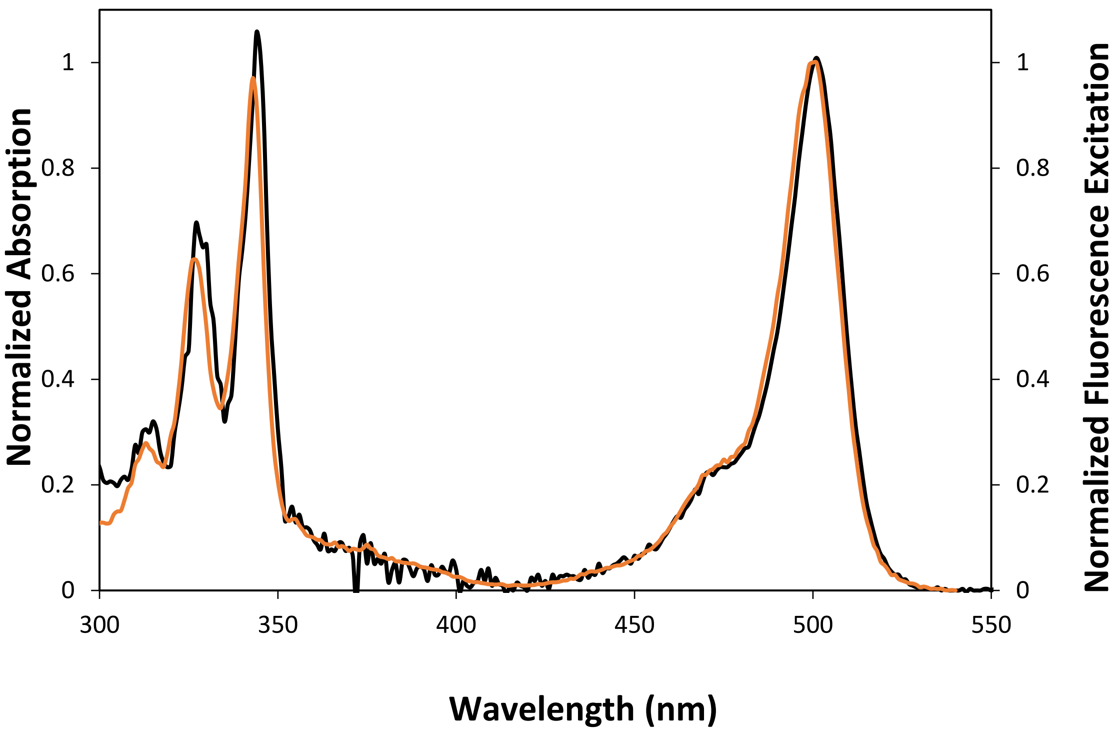


Figure S5: absorption (black line) and excitation (red line) spectra of compound 7
